# Integrating the interactome and the transcriptome of *Drosophila*

**DOI:** 10.1186/1471-2105-15-177

**Published:** 2014-06-10

**Authors:** Thilakam Murali, Svetlana Pacifico, Russell L Finley

**Affiliations:** 1 Center for Molecular Medicine and Genetics, Wayne State University School of Medicine, Detroit, Michigan 48201, USA; 2 Department of Biochemistry and Molecular Biology, Wayne State University School of Medicine, Detroit, Michigan 48201, USA

## Abstract

**Background:**

Networks of interacting genes and gene products mediate most cellular and developmental processes. High throughput screening methods combined with literature curation are identifying many of the protein-protein interactions (PPI) and protein-DNA interactions (PDI) that constitute these networks. Most of the detection methods, however, fail to identify the *in vivo* spatial or temporal context of the interactions. Thus, the interaction data are a composite of the individual networks that may operate in specific tissues or developmental stages. Genome-wide expression data may be useful for filtering interaction data to identify the subnetworks that operate in specific spatial or temporal contexts. Here we take advantage of the extensive interaction and expression data available for *Drosophila* to analyze how interaction networks may be unique to specific tissues and developmental stages.

**Results:**

We ranked genes on a scale from ubiquitously expressed to tissue or stage specific and examined their interaction patterns. Interestingly, ubiquitously expressed genes have many more interactions among themselves than do non-ubiquitously expressed genes both in PPI and PDI networks. While the PDI network is enriched for interactions between tissue-specific transcription factors and their tissue-specific targets, a preponderance of the PDI interactions are between ubiquitous and non-ubiquitously expressed genes and proteins. In contrast to PDI, PPI networks are depleted for interactions among tissue- or stage- specific proteins, which instead interact primarily with widely expressed proteins. In light of these findings, we present an approach to filter interaction data based on gene expression levels normalized across tissues or developmental stages. We show that this filter (the percent maximum or pmax filter) can be used to identify subnetworks that function within individual tissues or developmental stages.

**Conclusions:**

These observations suggest that protein networks are frequently organized into hubs of widely expressed proteins to which are attached various tissue- or stage-specific proteins. This is consistent with earlier analyses of human PPI data and suggests a similar organization of interaction networks across species. This organization implies that tissue or stage specific networks can be best identified from interactome data by using filters designed to include both ubiquitously expressed and specifically expressed genes and proteins.

## Background

The phenotypic identities of cells and tissues are governed in part by the particular regulatory networks that are active in them. Steady progress has been made to map the molecular interactions that constitute these networks, including the interactions among proteins and between transcription factors (TFs) and the genes that they regulate. Tens of thousands of protein-protein interactions (PPI) and TF-gene interactions have been identified for human and several model organisms, providing a foundation for identifying cell or tissue specific regulatory networks [1-4]. Most of the available interaction data, however, are noisy (i.e., include false positives and false negatives) and are derived from methods that are independent of the *in vivo* spatial or temporal context of the interactions. A majority of available PPI, for example, have come from two methods: the yeast two-hybrid system, which detects interactions between proteins expressed in a yeast nucleus [5-7], and protein complex determination [8,9], which usually involves forced expression of a tagged bait protein in a cultured cell line (for example, see [10,11]). The resulting data from these approaches can be used to build composite interactome networks representing many of the possible *in vivo* interactions. However, since only a fraction of these interactions may be active in a particular spatial or temporal context, filters are needed to identify the regulatory networks that are relevant to specific cells, tissues, or developmental time points.

To identify spatially or temporally relevant subnetworks, composite interactome networks can be filtered using gene expression or transcriptome data. It has been shown, for example, that interactions between proteins encoded by genes with similar or correlated expression patterns are more likely than those with dissimilar expression patterns to be genuine *in vivo* interactions [11-14]. This correlation can be used to predict new protein interactions [15,16], to score experimentally detected PPI [17-19], and to characterize different types of hub proteins within composite networks [20,21]. Correlated expression, however, is a relatively weak predictor of *in vivo* PPI and thus may not be useful for filtering interactome data to identify relevant subnetworks. An alternative approach would be to search the interactome data for subnetworks of genes that are specifically expressed in cells or tissues of interest. Recent studies on human PPI and transcriptome data, however, have suggested that tissue-specific proteins are involved in relatively few interactions, most of which are with house-keeping or ubiquitous proteins [22-24]. These studies suggested that tissue-specific proteins primarily interact with the more conserved ubiquitous proteins to modulate tissue-specific functions. If this were a general principle, filtering interaction data based on tissue-specific expression patterns would not be an effective method for identifying tissue-relevant subnetworks.

In this study we examined the relationship between the interactome and transcriptome of *Drosophila*. We took advantage of the extensive PPI and protein-DNA interaction (PDI) data available for *Drosophila*[25], and recent high quality transcriptome data for tissues [26] and developmental stages [27]. As suggested by the studies with human PPI data, we found that for *Drosophila*, tissue-specific proteins infrequently interacted among themselves but instead interacted primarily with widely expressed proteins. In addition, we show that stage-specific proteins have many more interactions with ubiquitously expressed proteins than with other stage-specific proteins. In contrast, we find that the *Drosophila* PDI network is enriched for interactions between tissue- and stage-specific TFs and their relevant tissue- and stage-specific targets, yet there is a preponderance of interactions between specifically expressed TFs and non-specifically expressed targets.

The specific interaction networks active in particular cells or tissues will be determined in part by the genes that are expressed in them. The problem of filtering an interactome network based on quantitative gene expression data, however, is particularly challenging because fully active genes can be expressed at widely different levels. In *Drosophila*, for example, mRNA abundances of different genes at their maximal level of expression can range over four orders of magnitude [26,27]. This problem, along with the finding that specifically expressed genes frequently interact with genes that are not expressed specifically, led us to develop a normalization procedure for gene expression data that takes into account the levels of expression across samples. The normalized expression value for a gene in a tissue or developmental stage is represented as the percentage of its maximum expression level across all tissues or stages, respectively. In order to identify networks that operate in different contexts, composite interactome networks can be filtered using this intuitive and quantitative gene expression filter. We show that the subnetworks identified with this filter are enriched for genes with mutant phenotypes that are relevant to different stages and tissues.

## Results and discussion

### Comparison of genes expressed ubiquitously or in specific tissues or developmental stages

To examine the interaction properties of *Drosophila* genes expressed in different patterns we classified genes based on the specificity of their expression across tissues or developmental stages (Methods). We used tissue expression data from FlyAtlas [26], which covers 15 adult and 8 larval tissues, and developmental stage expression data from the modENCODE project [27], which covers 30 developmental times points from embryo to adult. We classified genes expressed predominantly in one tissue as tissue specific (2838 genes), or in one stage as stage specific (3566 genes). We classified genes expressed across all tissues or all stages as tissue ubiquitous (3960 genes) or stage ubiquitous (4972 genes), respectively; 3226 of these genes are both tissue ubiquitous and stage ubiquitous and we refer to these as the common ubiquitous genes (Additional file 1). Overall, we classified 31% of the *Drosophila* genes as tissue ubiquitous and 22% as tissue-specific in this study. This is comparable to a study of human genes [23] in which 26% of the genes were classified as ubiquitous across 15 human tissues and cell lines and 13% were considered tissue-specific. As expected, the *Drosophila* common ubiquitous genes are enriched for genes involved in basic cellular processes, including protein synthesis, trafficking and degradation, RNA transcription and processing, and cytoskeleton organization (p values < 1 × 10^−8^). The ubiquitously expressed genes are also enriched for intracellular proteins (p values < 1 × 10^−8^) while the tissue- and stage-specific genes are enriched for extracellular proteins (p values < 5 × 10^−9^) (Methods). This is in partial agreement with the results of a human study where the tissue-specific proteins were found to be enriched for extracellular and membrane proteins [23]. The *Drosophila* ubiquitously expressed genes are also more evolutionarily conserved than the tissue- or stage-specific genes (Additional file 2). For example, about 37% of the ubiquitously expressed genes have yeast orthologs while only 12% of the tissue-specific genes and 8% of the stage-specific genes have yeast orthologs. Clear human orthologs exist for 80% of the ubiquitously expressed genes and only 19% and 14% of the tissue- and stage-specific genes, respectively. Among the genes that have orthologs in yeast or metazoans, about 50% are ubiquitously expressed while only 9-12% are tissue-specific and only 11-15% are stage-specific. This is in agreement with several studies examining the conservation of ubiquitously expressed and tissue-specific proteins in other organisms [28-32].

### Tissue- and stage-specific proteins predominantly interact with ubiquitously expressed proteins and not with each other

To determine the interaction properties of *Drosophila* proteins encoded by genes that are expressed ubiquitously or in specific tissues or stages we examined the 235,950 protein-protein interactions (PPI) available in the DroID database [25]. These include 93,544 interactions that were detected experimentally with *Drosophila* proteins, and a partially overlapping set of 144,171 potentially conserved interactions (interologs) that were predicted from experimental data in other species. For simplicity we refer to proteins encoded by ubiquitously expressed genes as “ubiquitous proteins”, and similarly to “tissue-specific proteins” and “stage-specific proteins”, keeping in mind that measured mRNA abundance may be an imperfect surrogate for protein abundance [33]. We found that the ubiquitous proteins have about three times more interactions per protein than the tissue- or stage-specific proteins (Table 1). When interologs are removed from the analysis (Table 1, numbers in parentheses), the ubiquitous proteins still have about three times more interactions than the tissue- or stage-specific proteins, indicating that the difference is not due simply to the fact that ubiquitous proteins are more conserved and therefore have more interactions that are interologs. This finding is in general agreement with findings for an analysis of human proteins in which proteins expressed in more tissues or cell lines had more interactions [23]. Next we looked at the numbers of interactions within and between the ubiquitous and the specific proteins. Roughly half of the protein interactions that involve ubiquitous proteins are with other ubiquitous proteins while only 12 – 15% of their interactions are with the specific proteins (Table 1). In contrast, fewer than 10% of the interactions involving the tissue- or stage-specific proteins are with other specifically expressed proteins, whereas around 60% of their interactions are with ubiquitous proteins (Table 1). Again, these differences were found independent of whether or not the interolog data was included. The finding that *Drosophila* tissue-specific proteins frequently interact with a core set of ubiquitously expressed proteins is consistent with analyses of the human interactome [22,23]. Combined, these results suggest that ubiquitous proteins frequently interact with each other while tissue- and stage-specific proteins frequently interact with widely expressed proteins and that this is a common feature of protein interaction networks identified in different species.

**Table 1:** Interactions involving ubiquitously and specifically expressed proteins

				**Percent of interactions with:**		
	**Proteins**	**Interactions/protein**	**Total interactions**	**Ubiquitous**	**NUNS**	**Specific**
Tissue ubiquitous	3960	37 (15)	148,849	49 (41)	37 (43)	14 (16)
Stage ubiquitous	4972	35 (14)	175,061	48 (46)	40 (37)	12 (16)
Tissue specific	2838	12 (6)	34,600	59 (54)	33 (40)	8 (7)
Stage specific	3566	10 (5)	37,897	56 (63)	35 (29)	9 (8)

Numbers are for all *Drosophila* protein interactions including those predicted from other species (interologs) but not measured with *Drosophila* proteins. Numbers in parentheses are for protein interactions detected experimentally with *Drosophila* proteins. NUNS are non-ubiquitous, non-specific proteins.

**Figure 1:**
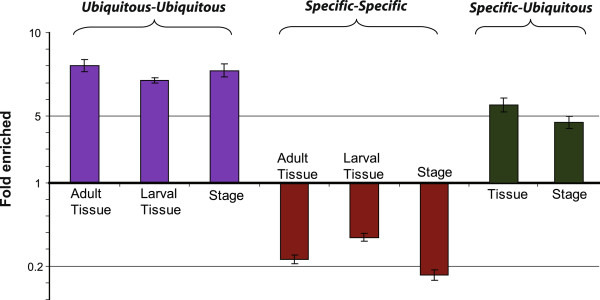
**Ubiquitous proteins frequently interact with each other while tissue- and stage- specific proteins interact with ubiquitous proteins more frequently than with each other.** For each set of proteins the number of interactions within the set (ubiquitous-ubiquitous and specific-specific) or between sets (ubiquitous-specific) was compared to the number of interactions in each of 5000 random protein sets taken from the composite PPI network. Each bar shows average fold-difference for 5000 trials. Standard deviations are shown as error bars. The p-value significance for each case is <7.56 × 10^-5^ at a CI = 99.99%. Ubiquitous-ubiquitous interactions were tested with adult tissue-ubiquitous proteins, larval tissue-ubiquitous proteins, and stage-ubiquitous proteins. Specific-specific interactions were tested with adult tissue-specific proteins, larval tissue-specific proteins, and stage-specific proteins. Ubiquitous-specific interactions were tested between adult tissue-ubiquitous and tissue-specific proteins or between stage-ubiquitous and stage-specific proteins.

The above analyses relied on counting interactions within and between groups of proteins, which may not be a good measure of the interaction tendencies of those groups. For example, if a group of proteins has few overall interactions (as do the specific proteins), then they will likely have few interactions among themselves just by chance. Likewise, proteins with many overall interactions are more likely to interact with each other just by chance. Moreover, since protein interaction networks generally follow a power law distribution of interactions per protein (degree) [34], the average degrees of groups of proteins may not be representative or useful for comparing between groups. Thus, to further compare the interactions of the ubiquitously and specifically expressed *Drosophila* proteins we calculated fold difference between the number of interactions among or between these groups and among random sets of other proteins of the same sizes (Methods). We found that both the tissue- and stage-ubiquitous proteins had about 8-fold (p-value 7.6 × 10^−5^) more interactions among themselves compared to other random proteins in the network (Figure 1). In contrast the tissue- and stage-specific PPI networks had about 5- to 6-fold (p-value 7.6 × 10^−5^) fewer interactions than the random proteins (Figure 1).

It is perhaps not surprising to find few interactions among all of the tissue- or stage-specific proteins, since many of them are never expressed together in the same tissue or at the same developmental stage. To determine whether the tissue- or stage- specific proteins interacted with each other within each tissue or stage, we built networks of genes expressed in each of the 23 adult and larval tissues and performed the same comparisons. The tissue-specific proteins within every tissue except the ovary had many fold fewer interactions among themselves than did random sets of other proteins expressed in the same tissue (Additional file 3A). Similarly, stage-specific proteins at each of the 30 developmental time points had relatively few interactions among themselves, with the exception of proteins specifically expressed in the one-day old female and the 6–8 hour embryo (Additional file 3B). The tissue- or stage-specific proteins rarely interacted among themselves even indirectly through third non-specific proteins (Additional file 4). We did the same analyses with larval tissue networks and obtained similar results (Additional file 5). Because the tissue- or stage-specific proteins rarely interacted with each other directly or indirectly, we determined whether they primarily interacted with the ubiquitous proteins. Both the tissue- and stage-specific proteins interacted about five-fold more with the ubiquitous network than did random sets of proteins (Figure 1). Thus, tissue- and stage-specific proteins generally do not form well-connected subnetworks but frequently interact with widely expressed proteins.

**Figure 2:**
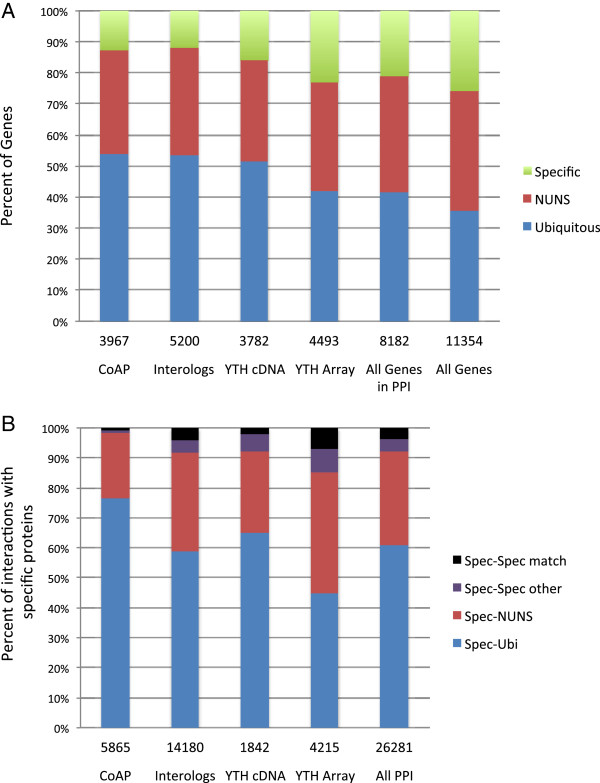
**Protein interaction data are biased toward ubiquitously expressed genes and interactions. A**. The fractions of proteins from the indicated interaction datasets that belong to each of three expression classes based on adult tissues: ubiquitous, non-ubiquitous non-specific (NUNS), and specific. **B**. The fraction of interactions involving tissue specific proteins that interact with ubiquitous, NUNS, or other tissue specific proteins. The tissue specific interactions include those from the same tissue (matched) or another tissue (other). Gene sets included all genes with tissue expression data (All Genes), and all genes with protein interactions in DroID (All Genes in PPI). Interaction data sets included data from yeast two-hybrid array screens (YTH Array), yeast two-hybrid cDNA screens (YTH cDNA), interologs from yeast, *C. elegans*, or human (Interologs), and protein interactions from co-affinity purification studies (coAP).

We considered the possibility that bias in the interaction data may contribute to the observation that ubiquitous proteins have more interactions than specific proteins. As shown in Figure 2A for adult tissues, all of the interaction data sets are biased toward tissue ubiquitous proteins. This is likely due to the fact that widely and highly expressed proteins are more readily identified in screens that have limited sensitivity, including mass spectrometry-based co-complex screens and yeast two-hybrid screens, the two methods accounting for the bulk of the interaction data. The bias toward ubiquitous proteins and against specific proteins does contribute to the numbers of interactions among these groups as illustrated in Figure 2B for tissue specific proteins. The dataset most biased toward ubiquitous proteins (Figure 2B, “co-AP”) has the highest frequency of interactions between specific and ubiquitous proteins and the lowest frequency of interactions between specific proteins, while the dataset with the least bias (Figure 2B, “Y2H array”) has the highest frequency of interactions among specific proteins. The least biased dataset, however, still shows a preponderance of interactions between the specifically expressed and the widely expressed proteins. Combined, these results suggest that tissue- or stage-specific proteins frequently interact with the ubiquitous core network. Thus, tissue- or stage-specific subnetworks must consist of a combination of specifically expressed proteins and widely expressed proteins. This further suggests that identification of tissue- or stage-specific networks could not be achieved by focusing only on tissue- or stage-specific protein interactions.

### Tissue- and stage-specific transcription factors frequently bind to ubiquitously expressed target genes in the protein-DNA interaction network

Next we determined the interaction tendencies of the ubiquitous and specific genes in the PDI. Not surprisingly, the ubiquitous TFs bound to ubiquitous targets more than expected by random chance (p-value 1.92 × 10^−5^). In addition, approximately half of the regulatory interactions of ubiquitous TFs involve non-ubiquitous targets. The PDI network is more limited than the PPI network because of the relatively small number of TFs that have been experimentally shown to bind target genes [35]. For each adult tissue there were only two or fewer tissue-specific TFs in the available PDI. Thus it is difficult to determine whether the tissue-specific TFs preferentially interact with the corresponding tissue-specific targets. Nevertheless, it is clear that a large fraction of the targets of tissue-specific TFs are not expressed exclusively in the same tissue. For example, the two testis-specific TFs in the PDI are involved in 562 interactions in the PDI and only 14 (2.5%) of these are with testis-specific targets. Likewise the single ovary- and heart-specific TFs in the PDI have 279 and 282 targets, only 7 (2.5%) and 3 (1.1%) of which are ovary- or heart-specific, respectively. In each case, most of the targets for tissue-specific TFs are expressed ubiquitously or in several tissues. We also examined a TF regulatory network that was predicted based on the integration of physical and functional interaction data [36]. Because one of the predictors for this network was developmental expression data, it would not be surprising to find enrichment for interactions between TFs and targets that are specifically expressed in the same patterns. Indeed, we found that the predicted network was from 1- to 9-fold enriched for interactions between adult tissue- or stage-specific TFs and targets that are specifically expressed in the corresponding tissue or stage (data not shown).

Overall, tissue-specific TFs interact with their corresponding tissue-specific targets about 3-fold more than expected from random chance. Thus, in contrast to the PPI network, the PDI network is enriched for interactions between TFs and targets that are expressed exclusively in the same tissue or stage (Additional file 6). Nevertheless, like the PPI network, in the PDI network most of the tissue-specific TF interactions are with non-specific targets. In the predicted PDI network, the 65 tissue-specific TFs are involved in 11,963 interactions, only 615 (5.1%) of which are with the corresponding tissue-specific targets. In contrast, 4355 (36.4%) of the interactions are with ubiquitously expressed targets, and most of the rest are with targets that we classified as neither tissue-specific nor ubiquitous; i.e., expressed in a subset of all tissues. Since the ubiquitous targets are by definition ubiquitously expressed, their regulation by specific TFs suggests that their levels are modulated in a context-dependent manner.

### An expression filter to identify genes active in specific contexts

Because the techniques used to collect much of the available interactome data are context independent, filters are needed to identify the subnetworks that operate in specific tissues or at specific developmental times. One type of filter that can be envisioned is one that retains only genes that are specifically expressed in a tissue or stage. However, as demonstrated above for *Drosophila* and elsewhere for human [22-24], proteins expressed in specific tissues or stages frequently interact with ubiquitously expressed proteins rather than with other specifically expressed proteins. In the PDI, ubiquitously expressed and specifically expressed genes are regulated by both types of TFs. Thus, a filter based on expression specificity is likely to remove many of the PPI or PDI interactions that are relevant in specific contexts. A second type of filter that can be envisioned is one that relies on absolute expression levels. For example, genes are frequently classified as “on” or “off” in a particular sample based on arbitrary expression thresholds. The problem with using filters based on expression thresholds, however, is that different genes may function at widely different expression levels; two genes, for example, may differ in their expression levels by several orders of magnitude even at their maximal levels.

We reasoned that a gene is more likely to be active when it is expressed at levels approaching its maximal level across all tissues or stages. For example, if a gene is maximally expressed in the ovary, it is likely to have a function in the ovary and in any other tissues where its level approaches the level in the ovary. To test this hypothesis we developed a scale to indicate the fraction of maximal expression for each gene in each tissue or developmental stage. For each tissue or stage we calculated a gene’s expression level as a percent of its level in the tissue or stage where it is maximally expressed. A gene, therefore, will have a percent maximum or “pmax” value for each tissue or stage.

To evaluate the pmax scale, we first asked if we could obtain gene lists enriched for tissue-relevant functions by selecting genes expressed above different pmax thresholds (e.g., pmax >50 or >75) in specific tissues. We compared these filters to one that selects genes expressed above the average value for all genes in a given tissue. We chose a set of six tissues (brain, thoracic ganglion, eye, testis, ovary, and larval central nervous system or CNS) and applied the three different filters to genes expressed in those tissues; the pmax filtered gene lists had in some cases more and in some cases fewer genes than the corresponding lists of genes expressed above the average level (Additional file 7). We evaluated the expression filters by checking for enrichment of tissue-relevant mutant phenotype annotations in the respective gene lists. The results show that the pmax filtered gene lists are highly enriched for tissue-relevant phenotypes in contrast to the gene lists filtered on average expression levels, which show little or no enrichment for tissue-relevant phenotypes (Additional file 8). The >75 pmax filter performed better than the >50 pmax filter for the selected tissues, supporting the hypothesis that genes expressed closer to their maximum level in a tissue are more likely to be functional in that tissue.

### Identification of context-relevant subnetworks

**Figure 3:**
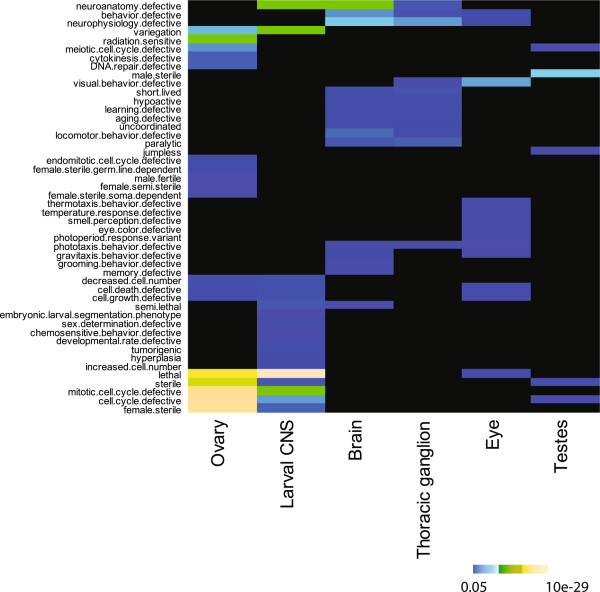
**Heat map of phenotypes enriched in six tissue-relevant subnetworks.** Heat map of enriched mutant phenotypes for genes in subnetworks containing only genes expressed above 75 pmax in ovary, larval CNS, brain, thoracic ganglion, testis, and eye. Corrected p-values for enrichment were log transformed, scaled (inset), and then plotted. Analysis for a complete list of phenotypes and tissues is shown in Additional file 9.

To test whether pmax values could be used to identify biologically relevant subnetworks in the PPI interactome we selected subnetworks containing interactions between genes expressed above 75 pmax in specific tissues and computed phenotype enrichment and depletion. The PPI subnetworks made from genes expressed above 75 pmax in specific tissues were highly enriched for tissue-relevant phenotypes (Figure 3 and Additional file 9). For example, the ovary subnetwork is enriched for the phenotype ‘female sterile’ (p-value 2.11 × 10^−26^), while the testis subnetwork is enriched for the phenotype ‘male sterile’ (p-value 1.32 × 10^−07^) (Figure 3). The brain and thoracic ganglion networks are enriched for phenotypes related to neuroanatomy, neurophysiology, and behavior, while the eye subnetwork is enriched for ‘visual behavior’ phenotypes (p-value 8.33 × 10^−06^). Likewise, many of the PPI subnetworks made from genes expressed above 75 pmax in specific stages were enriched for stage relevant phenotypes (Additional file 10). The early embryo networks, for example, were enriched for genes annotated with phenotypes related to the cell cycle, as expected given that early embryogenesis is dominated by cell division [37]. As development progresses from early embryo to late embryo, the subnetworks become enriched for behavioral phenotypes. As development progresses into larval and pupal stages and to adult, the subnetworks show little phenotype enrichment. This likely reflects the differentiation of postembryonic cells into collections of diverse tissues where it would be more appropriate to use tissue expression data to identify context-relevant subnetworks. The tissue and stage subnetworks with genes expressed at >75 pmax are also depleted for phenotypes that would not be expected for genes in specific tissues or stages (Additional files 11 and 12). For example, the subnetworks for ovary and early embryo stages are depleted of genes annotated with behavioral phenotypes. We also tested networks filtered at different pmax cut offs and found that generally, as the pmax cut off increased, the level of enrichment for genes with expected tissue-relevant phenotypes increased (Additional file 13). For example, networks with genes expressed >25 pmax or >45 pmax in the eye were enriched 1.8-fold or 2.5-fold for genes with the phenotype “visual behavior defective”, respectively (p-value 3.46 × 10^−03^ and p-value 7.63 × 10^−04^) (Additional file 13).

To further examine the effectiveness of the expression filter we asked whether the collection of enriched phenotypes in networks from each tissue or stage correlated with the collection of enriched phenotypes in related tissues or stages. We clustered the pmax-filtered subnetworks based on the similarity of phenotype terms that are enriched in them. In the case of the tissue-relevant pmax-filtered subnetworks (Additional file 9), the ovary and larval central nervous system clustered together as they are both enriched in genes that share ‘cell cycle’ and ‘lethality’ phenotypes among others. This is perhaps not surprising as it has been shown that many cell cycle and maternal genes play major roles in the asymmetric cell divisions in nervous system development [38,39]. Another example is the pmax PPI networks for brain and thoracic ganglion, which cluster together based on shared neuroanatomy and neurophysiology phenotypes among others. The testis subnetwork is an outgroup as it alone is enriched for the ‘male sterile’ phenotype, and similarly, the eye subnetwork is the only one enriched for phenotypes related to vision. Most larval tissue subnetworks cluster together due to their enrichment for lethality phenotypes. The early embryo subnetworks also cluster together, as do the late embryo subnetworks along with late pupal subnetworks (Additional file 10). The subnetwork at the mid-embryo stage from 10 to 12 hours forms an outgroup, consistent with studies [12,40] showing that transcripts specific for the early-embryo are down-regulated at this stage while late embryo-specific transcripts are just beginning to be expressed. The embryo subnetworks at 14–16 hrs and 16–18 hrs also do not cluster with the other embryo subnetworks and are not significantly enriched for embryo relevant phenotypes, corresponding to the transition to late embryo stages. It has been shown that related tissues show similar expression profiles and that tissues in consecutive developmental stages cluster together based on their gene expression patterns [41]. Here we show that related tissue pmax-filtered PPI subnetworks and as well as consecutive stage pmax-filtered PPI subnetworks cluster together based on related gene functions, as indicated by their shared mutant phenotypes. This result further shows that the pmax filter can identify subnetworks with the appropriate context relevant functions.

**Figure 4:**
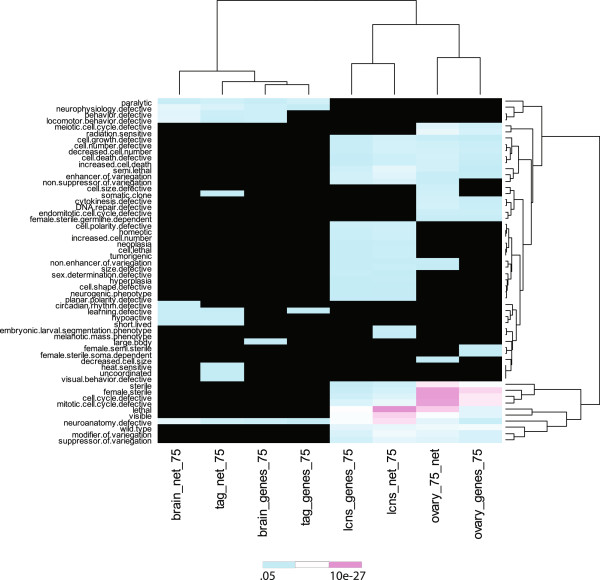
**Heat map of enriched phenotypes in 75 pmax filtered networks compared to 75 pmax filtered gene lists.** Heat map of enriched phenotypes obtained when genes expressed at 75 pmax or networks of genes expressed at 75 pmax in 4 different tissues were used as query genes and compared to random gene lists or networks. The p-values were log transformed, scaled and plotted.

We compared the phenotype enrichment of the 75 pmax subnetworks with similarly filtered gene lists. For this we picked the maximally expressed subnetworks and gene lists of the ovary, larval CNS, brain and thoracic ganglion (Figure 4). For example, applying a 75 pmax filter to the ovary results in 2502 genes expressed at >75 pmax expression level, while applying the same pmax filter to identify the ovary subnetwork results in a subnetwork with 2085 of these genes; to be maintained in the filtered subnetwork a gene expressed at >75 pmax must interact with another gene expressed at >75 pmax. As shown in Figure 4, the ovary subnetwork is more enriched than the ovary gene list for phenotypes such as ‘female sterile’, ‘mitotic cell cycle defective’, and ‘cell cycle defective’. Similar results were obtained with the other tissues (Figure 4) and with the PDI network (data not shown). Thus, pmax filtered subnetworks are better enriched for tissue-relevant phenotypes compared to the gene lists filtered at the same pmax. This is likely due to the fact that genes with related functions are frequently connected in the PPI and PDI subnetworks while genes with unrelated functions are more likely to be unconnected. Overall, these results suggest that the pmax filter is a useful method to identify the PPI and PDI subnetworks that operate in specific tissue contexts.

**Figure 5:**
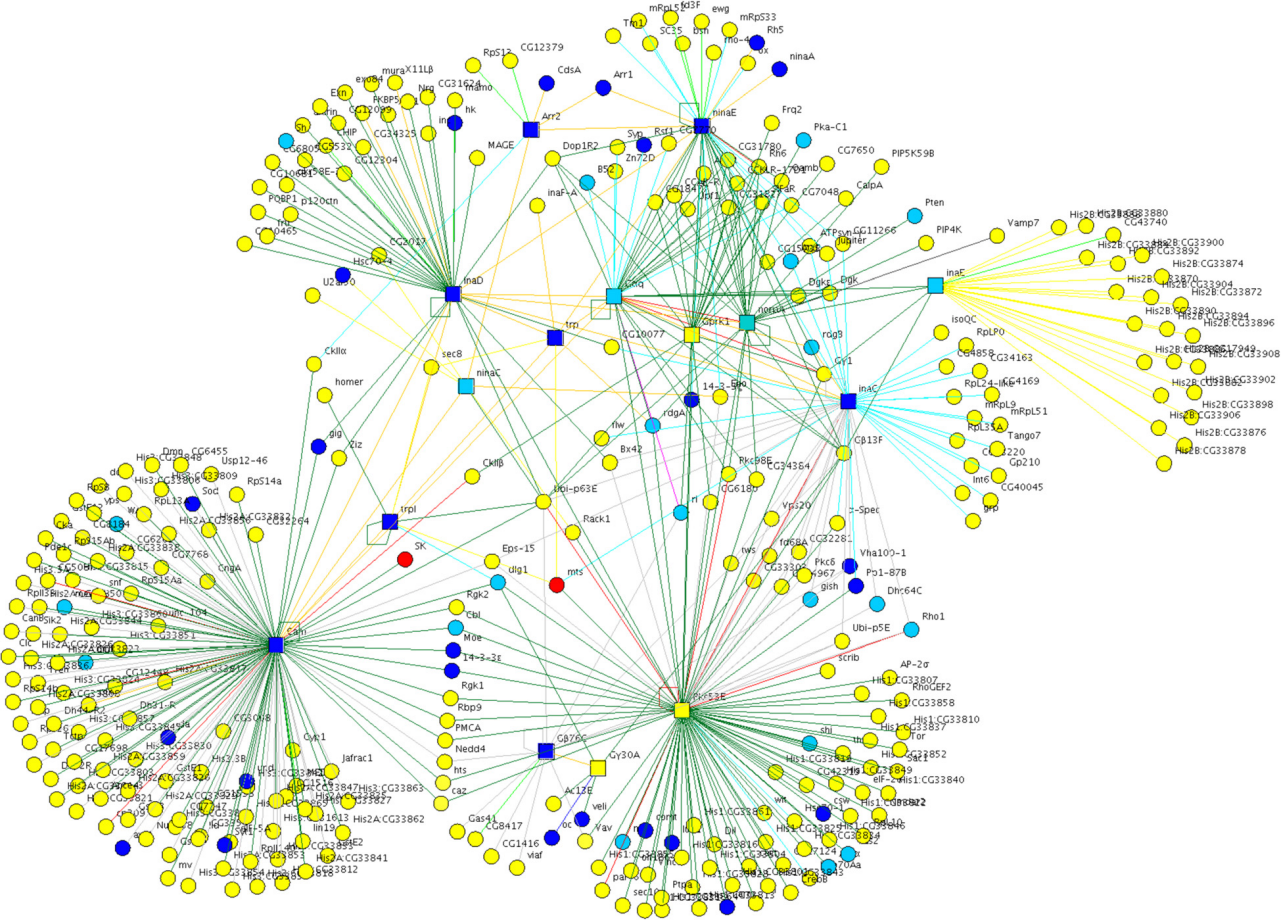
**An eye relevant network identified with the pmax expression filter.** The DroID database was searched for protein interactions involving genes in the KEGG pathway for phototransduction. The network was then filtered to remove genes expressed in the eye below 45% of their maximal value (pmax). The resulting network has 498 interactions among 351 proteins, including 15 of the KEGG pathway phototransduction proteins (squares) and 55 proteins encoded by genes known to have eye-related mutant phenotypes (see text). Among the proteins encoded by genes with eye-related phenotypes, 32 had tissue expression data (dark blue) and thus were subject to the pmax filter, while 23 had no expression data (light blue). After the initial analysis of phenotypes, examination of updated gene annotations and literature revealed an additional two genes with functions related to phototransduction (red). Edge colors reflect the source of the protein interaction data as described in Methods: dark green and yellow are human and *C. elegans* interologs, respectively; light blue and pink are from large co-complex studies; dark blue, green, and dark grey are from three separate large scale two-hybrid studies; orange are from literature databases; red are from more than one source. The pmax filter was set by default to retain genes that had no expression data. Deletion of genes with no expression data would result in a network further enriched for genes with eye related phenotypes, from 16.7% (58/348) to 20.3% (28/138); however, it would also eliminate many eye relevant genes, including genes with known eye related phenotypes that would no longer be connected to the network just because their expression is currently unknown.

To demonstrate how the pmax filter can be used to identify context-relevant pathways we generated a network for the phototransduction pathway that takes place in retinal cells (Figure 5). While several members of this pathway are well characterized, it is likely that additional members of the pathway remain to be discovered and that some of these novel members may be found in existing protein interaction data. The KEGG pathways database [42] lists 25 Drosophila genes involved in phototransduction, including genes for the major rhodopsin (Rh1), subunits of the heterotrimeric G-protein, Gq (Gß76C, Gα49B, Gγ30A), a phospholipase C or PLC (NorpA), diacyleglycerol kinase (inaE), and transient receptor potential (TRP) channels (TRP and TRPl). The signaling cascade is initiated when light promotes isomerization of a chromophore in rhodopsin leading to a conformational change that stimulates GDP-GTP exchange in Gq [43,44]. This stimulates the PLC to hydrolyze phosphatidylinositol 4,5-bisphosphate (PIP_2_) to diacylglycerol (DAG) and inositol 1,4,5-triphosphate (IP_3_), which in turn leads to opening of the TRP channels and influx of Ca^2+^. The pathway also includes genes for a protein kinase C or PKC (inaC), calmodulin (CaM), a G-protein-coupled receptor kinase (Gprk1), a protein that blocks the rhodopsin-Gq interactions called arrestin 2 (Arr2), a myosin III protein (ninaC), and a scaffold protein (inaD). We used these 25 genes to search the DroID database [25] and found protein interactions for all 25 KEGG phototransduction genes. The proteins are connected into a single protein interaction network containing 1186 proteins and 2655 interactions. At least some of these interactions, however, may be experimental artifacts, which are not uncommon in protein interaction data, while many other interactions may be biologically relevant in some contexts but have no role in phototransduction in the eye. To identify a subnetwork likely to contain additional phototransduction pathway proteins we filtered out genes expressed in the eye below 45% of their maximum value (pmax) across all tissues because we had shown that networks filtered for genes expressed >45 pmax are enriched for tissue-relevant genes (Additional file 13). This resulted in a network with 491 interactions among 348 proteins and included the 14 phototransduction genes named above plus another PKC (Pkc53E) (Figure 5). Among the KEGG phototransduction genes not included in the filtered network, only two are known to have eye-related functions, Act5C and ltp-r83A, and these are expressed in the eye at 37% and 27% of their tissue maximum, respectively. Most of the other KEGG phototransduction genes that were filtered out are only paralogs of the known phototransduction genes with as yet no demonstrated function in the eye. The network is enriched for genes that are annotated with one or more of the eye-related phenotype terms, including ‘photoreceptor’ (p < 6.007 × 10^−5^), ‘retina’ (p < 1.265 × 10^−6^), ‘visual behavior defective’ (p < 8.934 × 10^−10^), ‘phototaxis defective’ (p < 0.008) or ‘sensory perception defective’ (p < 0.01), in addition to ‘neuroanatomy defective’ (p < 8.021 × 10^−4^) and ‘neurophysiology defective’ (p < 1.335 × 10^−13^). In total 55 (16%) of the genes have eye-related phenotype terms, including 11 of the KEGG pathway genes (Figure 5 and Additional file 14). The significant enrichment for genes with known eye-related functions suggests that some of the poorly characterized genes in the network may also have eye-related functions. In support of this idea, a literature search revealed two additional genes in the network, SK and mts (Figure 5, red nodes) for which there is experimental evidence for phototransduction-related functions even though they were not yet annotated with eye phenotypes. SK is a small conductance calcium-activated potassium channel that has been shown to be required in photoreceptors for normal light response [45], while mts is a PP2A protein phosphatase that affects photoreceptor light adaptation by interacting with and dephosphorylating CaMKII [46].

**Figure 6:**
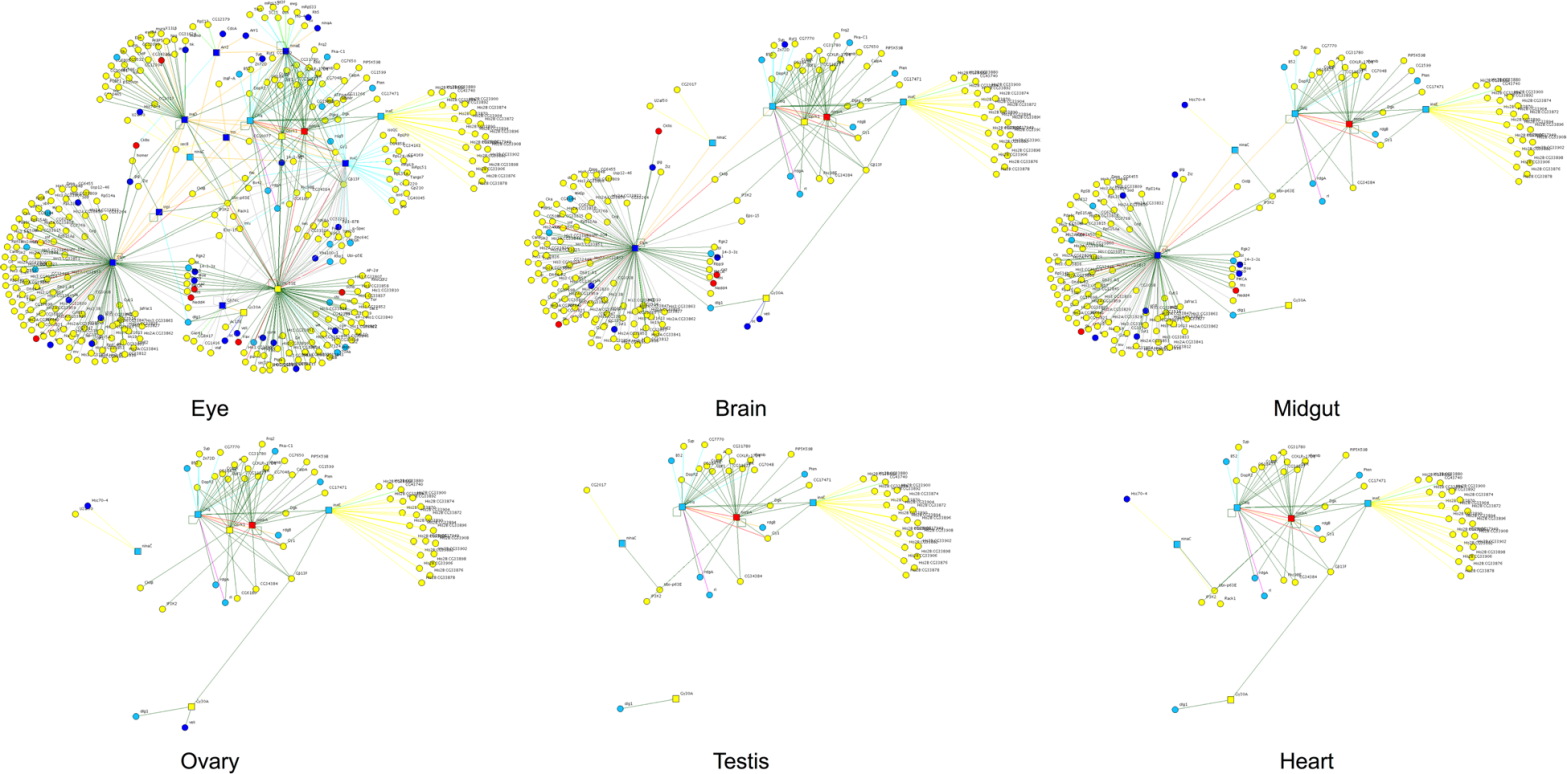
**Applying gene expression filters for different tissues disrupts the eye network.** The eye-relevant network (upper left) from Figure 5 was filtered to remove genes that are expressed below 45% pmax in the indicated tissues. The network filtered for brain expression is most similar to the original eye network, yet contains only about half of the proteins and interactions, only 7 of the 15 phototransduction proteins (squares), and less than half of the proteins encoded by genes with eye-related phenotypes. Many of the genes remaining in the filtered networks have no tissue expression data and thus could not have been removed by the expression filter. Colors are as in Figure 5.

Only 11 genes in the eye network have an eye-specific expression pattern. Thus, most of the genes in the network are expressed in a variety of tissues and could not have been identified by searching for genes with eye-specific expression. CaM, for example, is a ubiquitously expressed gene with a known role in phototransduction in the eye. While individual members of the pathway may be expressed in other tissues, we expect the network as a whole to be eye specific, since phototransduction occurs primarily in retinal cells. To test this we filtered out genes that were expressed below 45 pmax in each of several other tissues (Figure 6). This resulted in a striking disruption of the eye network, leaving behind mostly genes for which there is no data for tissue expression. Even the network filtered for brain expression, which was the one most similar to the original eye network, had only about half of the genes, interactions, genes with eye-related phenotypes, and KEGG phototransduction pathway genes. Combined, these results show that pmax expression values can be an effective filter to identify protein networks enriched for pathways that operate in specific tissues.

## Conclusions

In this study we used transcriptome data to examine the PPI and PDI interactomes of *Drosophila* and arrived at several general conclusions. First, ubiquitously expressed proteins interact among themselves significantly more than with specifically expressed proteins. Second, tissue- and stage-specific proteins interact with core networks of ubiquitously expressed proteins, potentially modifying them for tissue- or stage-specific functions. Third, we show that tissue- and stage-specific proteins rarely interact amongst themselves directly or even indirectly through other non-specific proteins. These results for PPI are in agreement with previous studies with human proteins showing that tissue-specific proteins have few interactions among themselves, ubiquitous proteins frequently interact with each other, and tissue-specific proteins primarily interact with ubiquitous proteins [22,23]. In addition, we have shown that these results hold true for developmental stage-specific and stage-ubiquitous proteins. The interactions of the tissue- or stage-specific proteins with the ubiquitous proteins may take place to recruit the ubiquitous network to perform context specific functions (some examples in [47-53]). In the PDI network, the tissue- and stage-specific TFs tend to regulate tissue- and stage-specific targets more than expected by random chance. A surprising finding in the PDI is that the tissue-specific TFs frequently regulate ubiquitous targets. The levels of expression of ubiquitous genes, therefore, may be regulated differently in specific tissues and stages, potentially rewiring the networks for specific functions.

These findings indicate that protein networks frequently consist of a core of evolutionarily conserved, widely-expressed proteins to which are attached a set of relatively new, tissue-specific proteins. Many of the functions of specific tissues may emerge via a modification of core non-specific cell networks. This is in contrast to the notion of cell- or tissue-specific systems that consist primarily of cell or tissue-specific proteins. The Drosophila phototransduction network (Figure 5) is a good example. Only 11 of the 351 genes in this network are specifically expressed in the eye. A large number of genes in the network that play a defined role in the eye, as indicated by their mutant phenotypes, are expressed in many different tissues in addition to the eye. An integral part of the phototransduction pathway, for example, is the calcium-signaling module, which is expressed and has a unique role in many different contexts [44,54].

The identification and characterization of cellular pathways and other functional modules is a major challenge in post-genome research. Clues about the constituents of pathways can be obtained from the huge amount of protein interaction data that is becoming available from high throughput screens and collections of data from published low throughput experiments. Since most of the available data comes from context-independent assays, many of the available interactions may not occur in any given biological setting. Gene expression information could be used as a first pass filter to identify interacting proteins that are expressed in the same cell or tissue. The finding that specifically expressed proteins frequently interact with ubiquitously expressed proteins shows that a filter based on tissue-specific expression would not be effective. Another implication of this finding is that genes encoding pathway members are not necessarily coordinately regulated across tissues. Thus, while correlated expression has been useful for finding groups of genes that may function together, the expression patterns of many members of specific pathways are not correlated. Another approach that has been used effectively in some studies is to first find genes that are expressed at high levels in a particular tissue in order to enrich for genes belonging to pathways that are active in that tissue (for example, [55]). Such an approach can generate false negatives, however, because some genes are active even when expressed at very low levels. As an example, the genes that are known to be required for Drosophila eye function (with eye-related mutant phenotypes) are expressed in the eye over a range of two orders of magnitude [26]. Many of the genes that are expressed at very low levels are transcription factors that are effectors in signal transduction pathways. Such genes would not be identified as potential members of eye-relevant pathways if expression levels were used to filter protein interaction data.

We set out to develop a method for using gene expression data to identify context-relevant networks from interactome data. We reasoned that a gene is likely to be active in a tissue where it is expressed at its maximal level relative to other tissues and that the closer a gene is to its maximal expression level the more likely it is to be active. The availability of quantitative gene expression data from a wide range of tissues or developmental stages has made it possible to test this idea. We used a normalization procedure that scaled (on a percent scale) each gene’s expression based on its maximum expression level in any tissue or stage. Each gene in each tissue or stage is expressed at a percent of its maximum value, or pmax. We showed that genes expressed at higher pmax values in a tissue or stage are more enriched for genes that function in that tissue. Moreover, filtering composite protein interaction networks using this scale generates biologically relevant subnetworks. Such a gene expression filter will be useful for generating hypotheses about the composition of pathways and other functional modules in cells. The pmax filter along with other gene expression filters should also be useful for understanding how the interactome changes from cell to cell and in different conditions (i.e., the dynamic interactome), particularly as additional expression data with better time and spatial resolution becomes available.

## Methods

### Interaction and expression data

Protein-protein interaction (PPI) and protein-DNA interaction (PDI) data were downloaded from DroID [14,25] version 2011_05, and include 93,544 experimentally detected PPI from yeast two-hybrid data from three large-scale studies [56-58] and an ongoing project [25], literature curated PPI from other major databases [59-61], and recently added PPI data from two large co-AP complex studies for *Drosophila*[10,62]. The PPI data also include 144,171 *Drosophila* interologs predicted from experimental data in yeast, worm and human [14,25]. In total we analyzed 235,950 unique PPI involving proteins from 10,823 genes. The analysis in Figure 2A and B used all unique, non-self PPI from DroID version 2014_01. Individual data sets included data from yeast two-hybrid array screens [56,58], yeast two-hybrid cDNA screens [56], interologs from yeast, *C. elegans*, or human [25], and protein interactions from co-affinity purification studies [10]. The *Drosophila* PDI data include 158,508 unique regulatory PDI for 149 transcription factors (TF) and 12,441 of their target genes that were inferred using TF binding and correlated expression of targets [35,63]. We separately analyzed ~300,000 computationally predicted PDI [36]. Tissue-wide gene expression data was downloaded from Flyatlas.org [26]. This data includes the mRNA signal that correlates with mRNA abundance for about 13,000 genes in 15 adult and 8 larval tissues measured using Affymetrix *Drosophila* expression arrays. Data was included for probes that mapped to single Flybase gene identifiers (FBgn). Probe data was included only where Affymetrix called the mRNA present for all four replicates. The mean signal from four replicates was used for all subsequent calculations. The stage-wide temporal gene expression data was obtained from Graveley *et al*. [27]. This data includes the mRNA abundance determined by RNA-Seq for more than 14,000 genes spanning 30 developmental time points from embryo to adult.

### Gene expression specificity scale

Genes were classified according to the specificity of their expression levels across all tissues or developmental stages. The expression specificity (*Es*_*i*_) of a gene in tissue or stage *i* is simply a fraction of the total abundance across all tissues or stages and was calculated as follows:

$$Es_{i}=\frac{\mathrm{abundanc}e_{i}}{\sum_{i=1}^{n}\mathrm{abundanc}e_{i}}$$

where *abundance*_*i*_ is the raw expression value of a gene in tissue or stage *i*. This results in *Es*_*i*_ values between 0 and 1 for each gene in each tissue or stage. The sum of all expression specificity values for each gene across all tissues or stages equals 1.

We placed genes into three non-overlapping bins based on their specificity of expression across all 15 adult tissues: Genes with *Es*_*i*_ values of > =0.8 in any of the adult tissues were labeled as tissue-specific (2838 genes); genes that were not tissue-specific and that had non-zero expression values across all adult tissues were labeled as ubiquitous (3960 genes); the remaining genes were labeled as tissue non-specific-non-ubiquitous (5830 genes). For the analyses in Additional file 5, we similarly classified genes using only the 8 larval tissues. In a separate classification, we placed genes into three non-overlapping bins based on their specificity of expression across 30 developmental stages: Genes with developmental stage *Es*_*i*_ values of > =0.19 were labeled as stage-specific (3566 genes); genes that were not stage-specific and that had developmental stage *Es*_*i*_ values >0.005 across all time points were labeled as ubiquitous (4972 genes); the remaining genes were labeled as stage non-ubiquitous, non-specific (6064 genes). The stage-specific bin included genes that showed a transient and sharp change in abundance that spanned a maximum of four consecutive developmental time points in a majority of the cases. The overlap among genes classified by the two specificity scales (tissue specificity and stage specificity) is shown in Additional file 1.

### Percent of maximum expression level (pmax) scale

We created a normalized gene expression scale to indicate for each gene the extent of its expression in a particular tissue or stage relative to its maximum level of expression in any tissue or stage, respectively. We refer to this normalized expression scale as the percent of maximum or pmax. To normalize gene expression based on this scale, the expression of a gene is calculated as the percent of its maximum expression level across all tissues or stages. For example, if a gene is maximally expressed in the ovary compared with all other tissues, then its pmax value in the mid-gut is its expression level in the midgut as a percentage of its expression in the ovary. A gene is expressed in tissue or stage *i* at a percent (*pmax*_*i*_) of its value in the tissue or stage where it is maximally expressed. *pmax*_*i*_ is calculated by:

$${\mathrm{pmax}}_{i}=\frac{\mathrm{abundanc}e_{i}}{\mathrm{abundanc}e_{\max}}\times \;100$$

where *abundance*_*i*_ is the raw expression value in tissue or stage *i*, and *abundance*_*max*_ is the raw expression value in the tissue or stage where it is maximally expressed. For the analysis in Figure 2, genes were similarly classified based only on adult tissues expression data. The pmax gene expression data and filters are available at the Drosophila interactions database, DroID (http://www.droidb.org) [25]. The database allows lists of PPI, PDI, and other interactions to be filtered based on user-defined pmax values. The pmax expression data can also be used to filter graphically displayed interaction networks using the interaction map browser tool (IM Browser) [64], which is also available at DroID. All precalculated pmax values for tissue and stage expression data are available for download at DroID.

### Orthology mapping

Potential *Drosophila* orthologs of yeast, worm and human proteins were identified using data downloaded from InParanoid version 7.0 database [65]. InParanoid performs pairwise comparisons of proteomes and constructs orthology groups. An orthology group has at least one protein from each species (seed orthologs) that are more similar to each other than to any other sequence in the other proteome. The orthology group may have additional sequences that are closer to the seed orthologs than to any sequences in the other proteome. We merged the InParanoid groups keeping a *Drosophila* gene as a unique reference to each orthology group.

### Network analyses

To compare the numbers of interactions among different groups of genes we calculated the fold difference in interactions over random expectation. First we counted the number of interactions among genes in the test set. Then we picked the same number of random genes from the PPI network minus the test set and counted interactions among them. The fold difference is the number of interactions among genes in the test set divided by the number of interactions among genes in the random set. We repeated this 5000 times for each test set. To calculate a p-value for each test case, we performed 100,000 Monte Carlo simulations by picking gene sets of the same size randomly and counting the number of interactions between the genes in each of these random sets. We computed the number of times the interactions in the random sets were lower, or higher, depending on the test case. This number was used to calculate the binomial confidence interval using binom.confint in R to calculate the p-value at a confidence of 99.99% (CI = 0.9999) [66,67]. We used the upper confidence interval as the p-value. Tissue-relevant networks were made from the composite network by including only the genes that had expression specificity values above zero in the respective tissues. Stage-relevant networks included only the genes that had expression specificity values above 0.005 in the respective stages. The PDI network is a directed network with a small number of TFs binding to numerous potentially regulated target genes. To find out if the tissue- and stage-specific TFs regulate tissue- and stage-specific targets more than expected by chance, for specific TFs in each tissue or stage we built random networks by assigning random targets while keeping the node degree constant. We built 100,000 such random networks for specific TFs in each of the tissues and stages. We computed the p-values (binom.confint, CI = 0.95) by counting the number of times specific interactions in the random networks were lower than the number of specific interactions in the tissue and stage PDI networks. Example networks were identified using IM Browser [64] to search and filter the DroID database [25]. Edge colors in Figures 5 and 6 depict the source of the protein interaction: dark green and yellow are human and *C. elegans* interologs, respectively; light blue and pink are from large co-complex studies [10,62]; dark blue, green, and dark grey are from three separate large scale two-hybrid studies [56-58]; orange are from literature databases [59-61]; red are from more than one source.

### Enrichment analyses

Gene Ontology enrichment analysis was performed using DAVID 6.7 [68] and the Drosophila protein-coding genes as background. We used DroPhEA [69] and BiNGO [70] to perform phenotype enrichment analysis. We used *Drosophila* phenotype controlled vocabulary terms from the FlyBase phenotype ontology [71]. The phenotypes resulting from single gene loci perturbations were used by DroPhEA and the same were used in the BiNGO analyses as background. About 4800 genes had phenotypes associated with them. The p-values were corrected by Bonferroni correction in DroPhEA and by Benjamini and Hochberg FDR correction in BiNGO and DAVID. The enriched and depleted p-values obtained for gene lists were negative log transformed and scaled (0–1) to create a distance matrix and then clustered hierarchically. R heatmap.plus package was used to plot the scaled values. We used BiNGO to compare phenotype enrichment in variously filtered gene lists and also to compare phenotype enrichment between filtered gene lists and filtered networks. We used DroPhEA to compare enrichment and depletion of phenotypes in different tissue and stage networks filtered for genes expressed at >75 pmax_i_.

### Availability of supporting data

All interaction data used in this study is available along with calculated gene expression pmax values in DroID, the Drosophila Interactions Database (http://www.droidb.org).

## Abbreviations

PDI: Protein-DNA interaction; TF: Transcription factor; PPI: Protein-protein interaction; pmax: Percent of maximum expression level.

## Competing interests

The authors declare that they have no competing interests.

## Authors’ contributions

TM assisted with the design of the study, carried out all analyses, prepared all figures and tables, and co-wrote the manuscript. SP assisted with network analysis and data accessibility; RLF conceived and designed the study, assisted with analyses, and co-wrote the manuscript. All authors read and approved the manuscript.

## Supplementary Material

Additional file 1 **Overlap of different groups of genes binned based on expression specificity.** Overlap of genes classified as tissue or stage specific, tissue or stage ubiquitous, and tissue or stage non- ubiquitous non-specific (NUNS). For tissue expression data only the adult tissues were used. Numbers in parentheses are total number of genes in each bin. Click here for file

Additional file 2 **Conservation of ubiquitously expressed genes.** Bars indicate the percentage of ubiquitous, tissue-specific, and stage-specific Drosophila genes that are conserved in each organism. Conservation is based on identification of close sequence homology (Methods). Click here for file

Additional file 3 **Within tissue- or stage-specific subnetworks, tissue- or stage-specific proteins rarely interact with each other.** For each protein set the average fold difference between the number of direct interactions in the test set and the number of interactions among other proteins in each of 5000 random sets of proteins expressed in the relevant tissue or stage is shown. Standard deviations are shown as error bars. The log p-values for each comparison are shown as red dots (right axis). (A) Interactions among the tissue-ubiquitous proteins or among each set of tissue-specific proteins in each of 15 adult tissues. (B) Interactions among the stage-ubiquitous proteins or among each set of stage-specific proteins for each of 30 developmental stages. Click here for file

Additional file 4 **Within tissue- or stage-specific subnetworks, tissue- or stage-specific proteins rarely interact with each other indirectly through a third protein.** For each protein set the average fold difference between the number of indirect interactions in the test set and number of indirect interactions among proteins in each of 5000 random sets of proteins expressed in the relevant tissue or stage is shown. Standard deviations are shown as error bars. The log p-values for each comparison are shown as red dots (right axis). > > indicates more than 200-fold less than random sets. (A) Indirect interactions among the tissue-ubiquitous proteins or among each set of tissue-specific proteins in each of 15 adult tissues. (B) Indirect interactions among the stage-ubiquitous proteins or among each set of stage-specific proteins for each of 30 developmental stages. Click here for file

Additional file 5 **Within larval tissue subnetworks, tissue-specific proteins rarely interact with each other directly or indirectly.** For each protein set the average fold difference between the number of direct (A) or indirect (B) interactions in the test set and number of direct or indirect interactions among proteins in each of 5000 random sets of proteins expressed in the relevant tissue is shown. Standard deviations are shown as error bars. The log p-values for each comparison are shown as red dots (right axis). (A) Direct interactions among the larval tissue-ubiquitous proteins or among each set of larval tissue-specific proteins. (B) Indirect interactions among each set of larval tissue-specific proteins. Click here for file

Additional file 6 **Tissue- and stage-specific transcription factors (TFs) regulate tissue- and stage-specific targets in the PDI networks more than expected by chance.** For specific TFs in each tissue or stage 100,000 random networks were built by assigning random targets while keeping the node degree constant. P-values (binom.confint, CI = 0.95) were computed by counting the number of times specific interactions in the random networks were lower than the number of specific interactions in the tissue and stage PDI networks. The protein-DNA interaction network (PDI) and the predicted PDI network (Methods) were analyzed separately. Click here for file

Additional file 7 **Number of genes in the tissue gene lists after applying different expression filters.** Number of genes expressed at average, greater than 50 pmax and greater than 75 pmax in six different tissues. Click here for file

Additional file 8 **Heat map of enriched phenotypes in variously filtered gene lists.** Heat map of enriched mutant phenotypes obtained in lists of genes because they were expressed above the average, greater than 50 pmax, or greater than 75 pmax in six different tissues. The corrected p-values for enrichment were log transformed, scaled, and then plotted. Click here for file

Additional file 9 **Heat map of phenotypes enriched in tissue-relevant subnetworks.** Heat map of enriched mutant phenotypes for genes in subnetworks containing only genes expressed above 75 pmax in each indicated tissue. The corrected p-values for enrichment were log transformed, scaled, and then plotted. Tissues are clustered based on the similarity of their enriched mutant phenotypes. Click here for file

Additional file 10 **Heat map of phenotypes enriched in stage-relevant subnetworks.** Heat map of enriched mutant phenotypes for genes in subnetworks containing only genes expressed above 75 pmax in each indicated stage. The corrected p-values for enrichment were log transformed, scaled, and then plotted. Stages are clustered based on the similarity of their enriched mutant phenotypes. Click here for file

Additional file 11 **Heat map of depleted phenotypes in tissue-relevant subnetworks.** Heat map of depleted mutant phenotypes for genes in subnetworks containing only genes expressed above 75 pmax in each indicated tissue. The corrected p-values for depletion were log transformed, scaled, and then plotted. Click here for file

Additional file 12 **Heat map of depleted phenotypes in stage-relevant subnetworks.** Mutant phenotypes for genes in subnetworks containing only genes expressed above 75 pmax in each indicated stage. The corrected p-values for depletion were log transformed, scaled, and then plotted. Click here for file

Additional file 13 **Networks filtered for genes expressed at higher pmax values are more enriched for context-relevant genes.** Top panel: shows fold enrichment for genes with the indicated phenotypes in protein interaction networks filtered for genes expressed above 25, 45, 65, or 85 pmax in brain (neurophysiology defective and behavior defective), ovary (female sterile), eye (visual behavior defective), or testis (male sterile). Fold enrichment is relative to the frequency of finding genes with those phenotypes in the unfiltered network. Bottom panel: Bonferroni-corrected p values for enrichment of the indicated phenotypes in the filtered networks relative to the frequency of those phenotypes in the proteome. Note that the unfiltered network is enriched for genes with some phenotypes, yet the enrichment increases with higher pmax filters. Click here for file

Additional file 14 **Filtered eye network interactions and genes.** List of all protein interactions in the filtered eye network in Figure 5. Genes are also listed along with their current function and phenotype annotations. Click here for file

## References

[B1] Braun P Gingras AC History of protein-protein interactions: from egg-white to complex networks Proteomics 2012 12 10 1478 1498 10.1002/pmic.201100563 22711592

[B2] Lemmens I Lievens S Tavernier J Strategies towards high-quality binary protein interactome maps J Proteomics 2010 73 8 1415 1420 10.1016/j.jprot.2010.02.001 20153845

[B3] Taylor IW Wrana JL Protein interaction networks in medicine and disease Proteomics 2012 12 10 1706 1716 10.1002/pmic.201100594 22593007

[B4] Ideker T Krogan NJ Differential network biology Mol Syst Biol 2012 8 565 22252388 10.1038/msb.2011.99 PMC3296360

[B5] Fields S Song O A novel genetic system to detect protein-protein interactions Nature 1989 340 6230 245 246 10.1038/340245a0 2547163

[B6] Venkatesan K Rual JF Vazquez A Stelzl U Lemmens I Hirozane-Kishikawa T Hao T Zenkner M Xin X Goh KI Yildirim MA Simonis N Heinzmann K Gebreab F Sahalie JM Cevik S Simon C de Smet AS Dann E Smolyar A Vinayagam A Yu H Szeto D Borick H Dricot A Klitgord N Murray RR Lin C Lalowski M Timm J An empirical framework for binary interactome mapping Nat Methods 2009 6 1 83 90 10.1038/nmeth.1280 19060904 PMC2872561

[B7] Parrish JR Gulyas KD Finley RL Jr Yeast two-hybrid contributions to interactome mapping Curr Opin Biotechnol 2006 17 4 387 393 10.1016/j.copbio.2006.06.006 16806892

[B8] Kocher T Superti-Furga G Mass spectrometry-based functional proteomics: from molecular machines to protein networks Nat Methods 2007 4 10 807 815 10.1038/nmeth1093 17901870

[B9] Gingras AC Aebersold R Raught B Advances in protein complex analysis using mass spectrometry J Physiol 2005 563 Pt 1 11 21 15611014 10.1113/jphysiol.2004.080440 PMC1665575

[B10] Guruharsha KG Rual JF Zhai B Mintseris J Vaidya P Vaidya N Beekman C Wong C Rhee DY Cenaj O McKillip E Shah S Stapleton M Wan KH Yu C Parsa B Carlson JW Chen X Kapadia B VijayRaghavan K Gygi SP Celniker SE Obar RA Artavanis-Tsakonas S A protein complex network of Drosophila melanogaster Cell 2011 147 3 690 703 10.1016/j.cell.2011.08.047 22036573 PMC3319048

[B11] Ewing RM Chu P Elisma F Li H Taylor P Climie S McBroom-Cerajewski L Robinson MD O'Connor L Li M Taylor R Dharsee M Ho Y Heilbut A Moore L Zhang S Ornatsky O Bukhman YV Ethier M Sheng Y Vasilescu J Abu-Farha M Lambert JP Duewel HS Stewart II Kuehl B Hogue K Colwill K Gladwish K Muskat B Large-scale mapping of human protein-protein interactions by mass spectrometry Mol Syst Biol 2007 3 89 17353931 10.1038/msb4100134 PMC1847948

[B12] Hooper SD Boue S Krause R Jensen LJ Mason CE Ghanim M White KP Furlong EE Bork P Identification of tightly regulated groups of genes during Drosophila melanogaster embryogenesis Mol Syst Biol 2007 3 72 17224916 10.1038/msb4100112 PMC1800352

[B13] Lee I Date SV Adai AT Marcotte EM A probabilistic functional network of yeast genes Science 2004 306 5701 1555 1558 10.1126/science.1099511 15567862

[B14] Yu J Pacifico S Liu G Finley RL Jr DroID: the Drosophila Interactions Database, a comprehensive resource for annotated gene and protein interactions BMC Genomics 2008 9 461 10.1186/1471-2164-9-461 18840285 PMC2572628

[B15] Jansen R Yu H Greenbaum D Kluger Y Krogan NJ Chung S Emili A Snyder M Greenblatt JF Gerstein M A Bayesian networks approach for predicting protein-protein interactions from genomic data Science 2003 302 5644 449 453 10.1126/science.1087361 14564010

[B16] Scott MS Barton GJ Probabilistic prediction and ranking of human protein-protein interactions BMC Bioinformatics 2007 8 239 10.1186/1471-2105-8-239 17615067 PMC1939716

[B17] Yu J Finley RL Jr Combining multiple positive training sets to generate confidence scores for protein-protein interactions Bioinformatics 2009 25 1 105 111 10.1093/bioinformatics/btn597 19010802 PMC2638943

[B18] Karagoz K Arga KY Assessment of high-confidence protein-protein interactome in yeast Comput Biol Chem 2013 45 1 8 23608186 10.1016/j.compbiolchem.2013.03.002

[B19] Li D Liu W Liu Z Wang J Liu Q Zhu Y He F PRINCESS, a protein interaction confidence evaluation system with multiple data sources Mol Cell Proteomics 2008 7 6 1043 1052 10.1074/mcp.M700287-MCP200 18230642

[B20] Han JD Bertin N Hao T Goldberg DS Berriz GF Zhang LV Dupuy D Walhout AJ Cusick ME Roth FP Evidence for dynamically organized modularity in the yeast protein-protein interaction network Nature 2004 430 6995 88 93 10.1038/nature02555 15190252

[B21] Kim PM Lu LJ Xia Y Gerstein MB Relating three-dimensional structures to protein networks provides evolutionary insights Science 2006 314 5807 1938 1941 10.1126/science.1136174 17185604

[B22] Bossi A Lehner B Tissue specificity and the human protein interaction network Mol Syst Biol 2009 5 260 19357639 10.1038/msb.2009.17 PMC2683721

[B23] Emig D Albrecht M Tissue-specific proteins and functional implications J Proteome Res 2011 10 4 1893 1903 10.1021/pr101132h 21341747

[B24] Lin WH Liu WC Hwang MJ Topological and organizational properties of the products of house-keeping and tissue-specific genes in protein-protein interaction networks BMC Syst Biol 2009 3 32 10.1186/1752-0509-3-32 19284572 PMC2663781

[B25] Murali T Pacifico S Yu J Guest S Roberts GG 3rd Finley RL Jr DroID 2011: a comprehensive, integrated resource for protein, transcription factor, RNA and gene interactions for Drosophila Nucleic Acids Res 2011 39 Database issue D736 743 21036869 10.1093/nar/gkq1092 PMC3013689

[B26] Chintapalli VR Wang J Dow JA Using FlyAtlas to identify better Drosophila melanogaster models of human disease Nat Genet 2007 39 6 715 720 10.1038/ng2049 17534367

[B27] Graveley BR Brooks AN Carlson JW Duff MO Landolin JM Yang L Artieri CG van Baren MJ Boley N Booth BW Brown JB Cherbas L Davis CA Dobin A Li R Lin W Malone JH Mattiuzzo NR Miller D Sturgill D Tuch BB Zaleski C Zhang D Blanchette M Dudoit S Eads B Green RE Hammonds A Jiang L Kapranov P The developmental transcriptome of Drosophila melanogaster Nature 2011 471 7339 473 479 10.1038/nature09715 21179090 PMC3075879

[B28] Lehner B Fraser AG Protein domains enriched in mammalian tissue-specific or widely expressed genes Trends Genet 2004 20 10 468 472 10.1016/j.tig.2004.08.002 15363898

[B29] Winter EE Goodstadt L Ponting CP Elevated rates of protein secretion, evolution, and disease among tissue-specific genes Genome Res 2004 14 1 54 61 14707169 10.1101/gr.1924004 PMC314278

[B30] Zhang L Li WH Mammalian housekeeping genes evolve more slowly than tissue-specific genes Mol Biol Evol 2004 21 2 236 239 14595094 10.1093/molbev/msh010

[B31] Freilich S Massingham T Bhattacharyya S Ponsting H Lyons PA Freeman TC Thornton JM Relationship between the tissue-specificity of mouse gene expression and the evolutionary origin and function of the proteins Genome Biol 2005 6 7 R56 10.1186/gb-2005-6-7-r56 15998445 PMC1175987

[B32] Tu Z Wang L Xu M Zhou X Chen T Sun F Further understanding human disease genes by comparing with housekeeping genes and other genes BMC Genomics 2006 7 31 10.1186/1471-2164-7-31 16504025 PMC1397819

[B33] Maier T Guell M Serrano L Correlation of mRNA and protein in complex biological samples FEBS Lett 2009 583 24 3966 3973 10.1016/j.febslet.2009.10.036 19850042

[B34] Barabasi AL Oltvai ZN Network biology: understanding the cell’s functional organization Nat Rev Genet 2004 5 2 101 113 10.1038/nrg1272 14735121

[B35] Roy S Ernst J Kharchenko PV Kheradpour P Negre N Eaton ML Landolin JM Bristow CA Ma L Lin MF Washietl S Arshinoff BI Ay F Meyer PE Robine N Washington NL Di Stefano L Berezikov E Brown CD Candeias R Carlson JW Carr A Jungreis I Marbach D Sealfon R Tolstorukov MY Will S Alekseyenko AA Artieri C Booth BW Identification of functional elements and regulatory circuits by Drosophila modENCODE Science 2010 330 6012 1787 1797 21177974 10.1126/science.1198374 PMC3192495

[B36] Marbach D Roy S Ay F Meyer PE Candeias R Kahveci T Bristow CA Kellis M Predictive regulatory models in Drosophila melanogaster by integrative inference of transcriptional networks Genome Res 2012 22 7 1334 1349 10.1101/gr.127191.111 22456606 PMC3396374

[B37] Edgar BA Lehner CF Developmental control of cell cycle regulators: a fly’s perspective Science 1996 274 5293 1646 1652 10.1126/science.274.5293.1646 8939845

[B38] Chia W Somers WG Wang H Drosophila neuroblast asymmetric divisions: cell cycle regulators, asymmetric protein localization, and tumorigenesis J Cell Biol 2008 180 2 267 272 10.1083/jcb.200708159 18209103 PMC2213578

[B39] Hafer N Xu S Bhat KM Schedl P The Drosophila CPEB protein Orb2 has a novel expression pattern and is important for asymmetric cell division and nervous system function Genetics 2011 189 3 907 921 10.1534/genetics.110.123646 21900268 PMC3213381

[B40] Kalinka AT Varga KM Gerrard DT Preibisch S Corcoran DL Jarrells J Ohler U Bergman CM Tomancak P Gene expression divergence recapitulates the developmental hourglass model Nature 2010 468 7325 811 814 10.1038/nature09634 21150996

[B41] Li S Pandey S Gookin TE Zhao Z Wilson L Assmann SM Gene-sharing networks reveal organizing principles of transcriptomes in Arabidopsis and other multicellular organisms Plant Cell 2012 24 4 1362 1378 10.1105/tpc.111.094748 22517316 PMC3398552

[B42] Kotera M Hirakawa M Tokimatsu T Goto S Kanehisa M The KEGG databases and tools facilitating omics analysis: latest developments involving human diseases and pharmaceuticals Methods Mol Biol 2012 802 19 39 10.1007/978-1-61779-400-1_2 22130871

[B43] Katz B Minke B Drosophila photoreceptors and signaling mechanisms Front Cell Neurosci 2009 3 2 19623243 10.3389/neuro.03.002.2009 PMC2701675

[B44] Montell C Drosophila visual transduction Trends Neurosci 2012 35 6 356 363 10.1016/j.tins.2012.03.004 22498302 PMC3367115

[B45] Abou Tayoun AN Li X Chu B Hardie RC Juusola M Dolph PJ The Drosophila SK channel (dSK) contributes to photoreceptor performance by mediating sensitivity control at the first visual network J Neurosci 2011 31 39 13897 13910 10.1523/JNEUROSCI.3134-11.2011 21957252 PMC3758547

[B46] Lu H Leung HT Wang N Pak WL Shieh BH Role of Ca2+/calmodulin-dependent protein kinase II in Drosophila photoreceptors J Biol Chem 2009 284 17 11100 11109 10.1074/jbc.M806956200 19254957 PMC2670115

[B47] Uechi T Nakajima Y Nakao A Torihara H Chakraborty A Inoue K Kenmochi N Ribosomal protein gene knockdown causes developmental defects in zebrafish PLoS One 2006 1 e37 10.1371/journal.pone.0000037 17183665 PMC1762390

[B48] Raices M D’Angelo MA Nuclear pore complex composition: a new regulator of tissue-specific and developmental functions Nat Rev Mol Cell Biol 2012 13 11 687 699 10.1038/nrm3461 23090414

[B49] Bowne SJ Liu Q Sullivan LS Zhu J Spellicy CJ Rickman CB Pierce EA Daiger SP Why do mutations in the ubiquitously expressed housekeeping gene IMPDH1 cause retina-specific photoreceptor degeneration? Invest Ophthalmol Vis Sci 2006 47 9 3754 3765 10.1167/iovs.06-0207 16936083 PMC2581456

[B50] Weake VM Dyer JO Seidel C Box A Swanson SK Peak A Florens L Washburn MP Abmayr SM Workman JL Post-transcription initiation function of the ubiquitous SAGA complex in tissue-specific gene activation Genes Dev 2011 25 14 1499 1509 10.1101/gad.2046211 21764853 PMC3143940

[B51] Weake VM Workman JL SAGA function in tissue-specific gene expression Trends Cell Biol 2011 22 4 177 184 22196215 10.1016/j.tcb.2011.11.005 PMC3322277

[B52] Hiller M Chen X Pringle MJ Suchorolski M Sancak Y Viswanathan S Bolival B Lin TY Marino S Fuller MT Testis-specific TAF homologs collaborate to control a tissue-specific transcription program Development 2004 131 21 5297 5308 10.1242/dev.01314 15456720

[B53] Matzat LH Dale RK Moshkovich N Lei EP Tissue-specific regulation of chromatin insulator function PLoS Genet 2012 8 11 e1003069 10.1371/journal.pgen.1003069 23209434 PMC3510032

[B54] Clapham DE Calcium signaling Cell 2007 131 6 1047 1058 10.1016/j.cell.2007.11.028 18083096

[B55] Blackshaw S Fraioli RE Furukawa T Cepko CL Comprehensive analysis of photoreceptor gene expression and the identification of candidate retinal disease genes Cell 2001 107 5 579 589 10.1016/S0092-8674(01)00574-8 11733058

[B56] Giot L Bader JS Brouwer C Chaudhuri A Kuang B Li Y Hao YL Ooi CE Godwin B Vitols E Vijayadamodar G Pochart P Machineni H Welsh M Kong Y Zerhusen B Malcolm R Varrone Z Collis A Minto M Burgess S McDaniel L Stimpson E Spriggs F Williams J Neurath K Ioime N Agee M Voss E Furtak K A protein interaction map of Drosophila melanogaster Science 2003 302 5651 1727 1736 10.1126/science.1090289 14605208

[B57] Formstecher E Aresta S Collura V Hamburger A Meil A Trehin A Reverdy C Betin V Maire S Brun C Jacq B Arpin M Bellaiche Y Bellusci S Benaroch P Bornens M Chanet R Chavrier P Delattre O Doye V Fehon R Faye G Galli T Girault JA Goud B de Gunzburg J Johannes L Junier MP Mirouse V Mukherjee A Protein interaction mapping: a Drosophila case study Genome Res 2005 15 3 376 384 10.1101/gr.2659105 15710747 PMC551564

[B58] Stanyon CA Liu G Mangiola BA Patel N Giot L Kuang B Zhang H Zhong J Finley RL Jr A Drosophila protein-interaction map centered on cell-cycle regulators Genome Biol 2004 5 12 R96 10.1186/gb-2004-5-12-r96 15575970 PMC545799

[B59] Ceol A Chatr Aryamontri A Licata L Peluso D Briganti L Perfetto L Castagnoli L Cesareni G MINT, the molecular interaction database: 2009 update Nucleic Acids Res 2010 38 Database issue D532 539 19897547 10.1093/nar/gkp983 PMC2808973

[B60] Chatr-Aryamontri A Breitkreutz BJ Heinicke S Boucher L Winter A Stark C Nixon J Ramage L Kolas N O’Donnell L Reguly T Breitkreutz A Sellam A Chen D Chang C Rust J Livstone M Oughtred R Dolinski K Tyers M The BioGRID interaction database: 2013 update Nucleic Acids Res 2013 41 (Database issue) D816 23 23203989 10.1093/nar/gks1158 PMC3531226

[B61] Kerrien S Aranda B Breuza L Bridge A Broackes-Carter F Chen C Duesbury M Dumousseau M Feuermann M Hinz U Jandrasits C Jimenez RC Khadake J Mahadevan U Masson P Pedruzzi I Pfeiffenberger E Porras P Raghunath A Roechert B Orchard S Hermjakob H The IntAct molecular interaction database in 2012 Nucleic Acids Res 2012 40 Database issue D841 846 22121220 10.1093/nar/gkr1088 PMC3245075

[B62] Friedman AA Tucker G Singh R Yan D Vinayagam A Hu Y Binari R Hong P Sun X Porto M Pacifico S Murali T Finley RL Jr Asara JM Berger B Perrimon N Proteomic and functional genomic landscape of receptor tyrosine kinase and ras to extracellular signal-regulated kinase signaling Sci Signal 2011 4 196 rs10 22028469 10.1126/scisignal.2002029 PMC3439136

[B63] Gallo SM Gerrard DT Miner D Simich M Des Soye B Bergman CM Halfon MS REDfly v3.0: toward a comprehensive database of transcriptional regulatory elements in Drosophila Nucleic Acids Res 2011 39 Database issue D118 123 20965965 10.1093/nar/gkq999 PMC3013816

[B64] Pacifico S Liu G Guest S Parrish JR Fotouhi F Finley RL Jr A database and tool, IM Browser, for exploring and integrating emerging gene and protein interaction data for Drosophila BMC Bioinformatics 2006 7 195 10.1186/1471-2105-7-195 16603075 PMC1458360

[B65] Ostlund G Schmitt T Forslund K Kostler T Messina DN Roopra S Frings O Sonnhammer EL InParanoid 7: new algorithms and tools for eukaryotic orthology analysis Nucleic Acids Res 2010 38 Database issue D196 203 19892828 10.1093/nar/gkp931 PMC2808972

[B66] Ott J Analysis of human genetic linkage 1999 3 Baltimore, Maryland: The Johns Hopkins University Press

[B67] Snedecor GW Cochran WG Statistical methods 1989 8 Ames, Iowa: Iowa State University Press

[B68] da Huang W Sherman BT Lempicki RA Systematic and integrative analysis of large gene lists using DAVID bioinformatics resources Nat Protoc 2009 4 1 44 57 19131956 10.1038/nprot.2008.211

[B69] Weng MP Liao BY DroPhEA: Drosophila phenotype enrichment analysis for insect functional genomics Bioinformatics 2011 27 22 3218 3219 10.1093/bioinformatics/btr530 21976423

[B70] Maere S Heymans K Kuiper M BiNGO: a Cytoscape plugin to assess overrepresentation of gene ontology categories in biological networks Bioinformatics 2005 21 16 3448 3449 10.1093/bioinformatics/bti551 15972284

[B71] McQuilton P St Pierre SE Thurmond J FlyBase 101–the basics of navigating FlyBase Nucleic Acids Res 2012 40 Database issue D706 714 22127867 10.1093/nar/gkr1030 PMC3245098

