# Supplementary material for: Integrating the interactome and the transcriptome of Drosophila
Source: BMC Bioinformatics. 2014 Jun 10;15:177. doi: 10.1186/1471-2105-15-177 (PMC4229734; doi:10.1186/1471-2105-15-177)
Supplement: Additional file 1 — Overlap of different groups of genes binned based on expression specificity. Overlap of genes classified as tissue or stage specific, tissue or stage ubiquitous, and tissue or stage non- ubiquitous non-specific (NUNS). For tissue expression data only the adult tissues were used. Numbers in parentheses are total number of genes in each bin. [file 1471-2105-15-177-S1.pdf]

	adult tissue-specific (2838)	adult tissue-NUNS (5830)	adult tissue-ubiquitous (3960)
**stage-specific (3566)**	1363	1546	41
**stage-NUNS (6064)**	1353	2419	536
**stage-ubiquitous (4972)**	49	1290	3226
