# Supplementary figures and images for: Integrating the interactome and the transcriptome of Drosophila
Source: BMC Bioinformatics. 2014 Jun 10;15:177. doi: 10.1186/1471-2105-15-177 (PMC4229734; doi:10.1186/1471-2105-15-177)

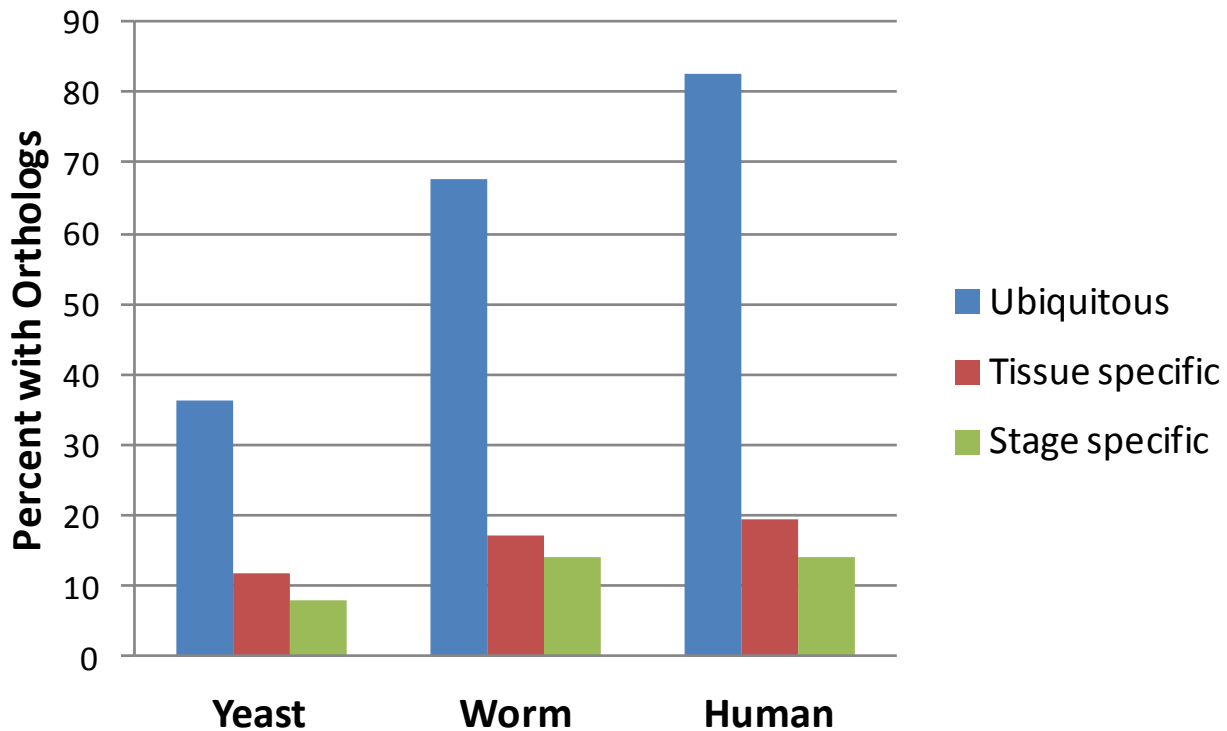

Supplement: Additional file 2 — Conservation of ubiquitously expressed genes. Bars indicate the percentage of ubiquitous, tissue-specific, and stage-specific Drosophila genes that are conserved in each organism. Conservation is based on identification of close sequence homology (Methods). [file 1471-2105-15-177-S2.pdf]

A

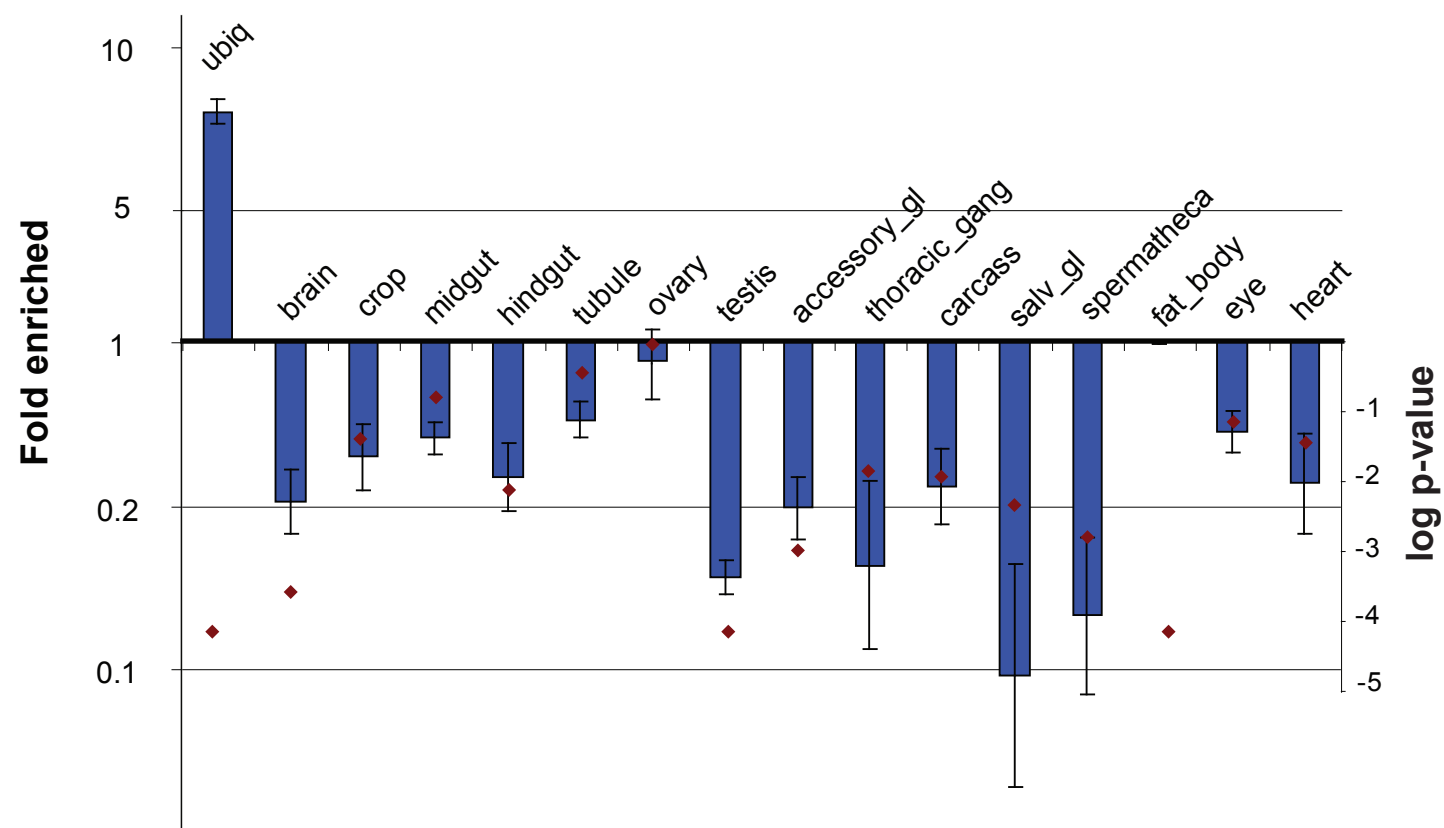

B

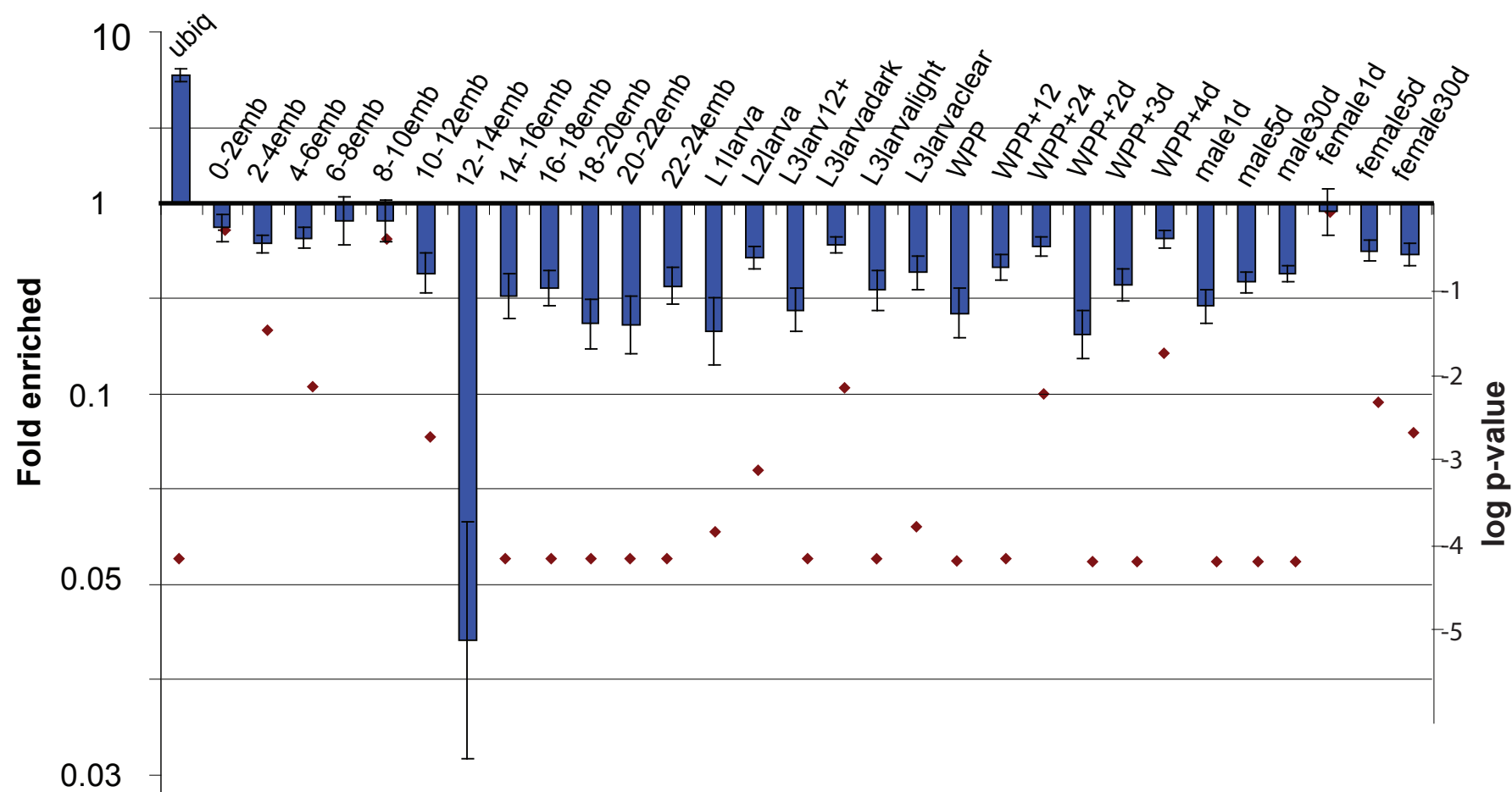

Supplement: Additional file 3 — Within tissue- or stage-specific subnetworks, tissue- or stage-specific proteins rarely interact with each other. For each protein set the average fold difference between the number of direct interactions in the test set and the number of interactions among other proteins in each of 5000 random sets of proteins expressed in the relevant tissue or stage is shown. Standard deviations are shown as error bars. The log p-values for each comparison are shown as red dots (right axis). (A) Interactions among the tissue-ubiquitous proteins or among each set of tissue-specific proteins in each of 15 adult tissues. (B) Interactions among the stage-ubiquitous proteins or among each set of stage-specific proteins for each of 30 developmental stages. [file 1471-2105-15-177-S3.pdf]

A

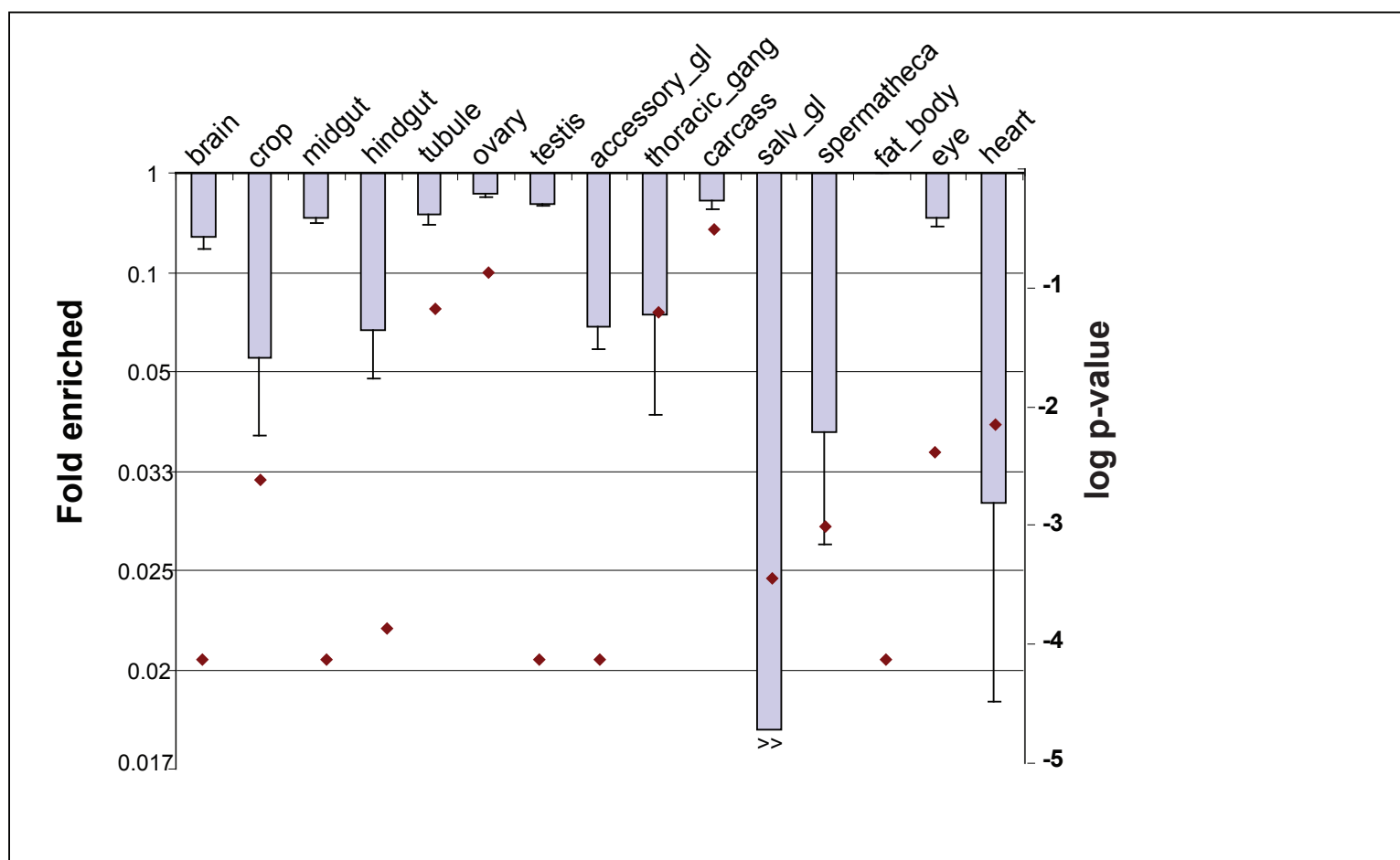

B

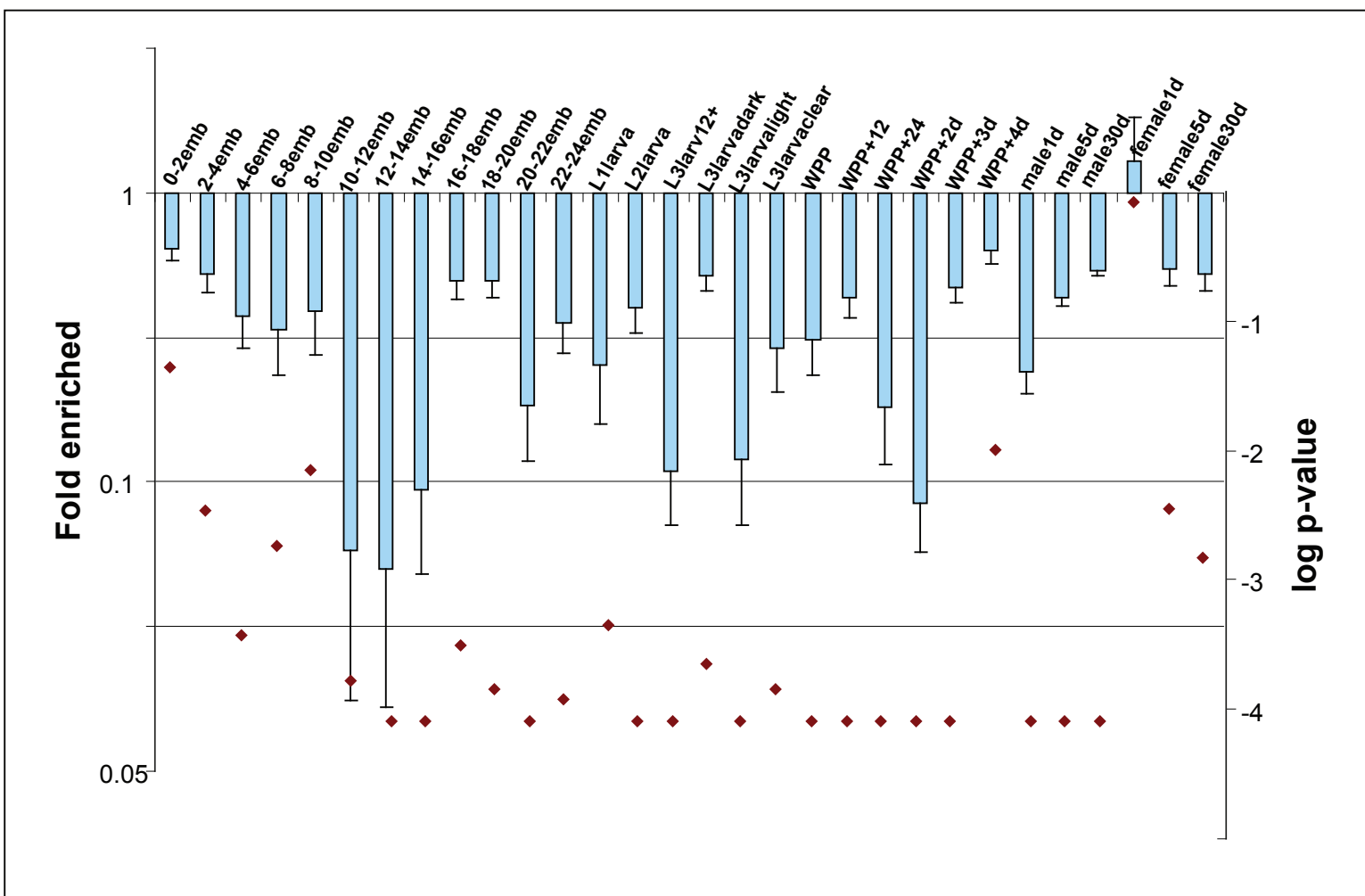

Supplement: Additional file 4 — Within tissue- or stage-specific subnetworks, tissue- or stage-specific proteins rarely interact with each other indirectly through a third protein. For each protein set the average fold difference between the number of indirect interactions in the test set and number of indirect interactions among proteins in each of 5000 random sets of proteins expressed in the relevant tissue or stage is shown. Standard deviations are shown as error bars. The log p-values for each comparison are shown as red dots (right axis). > > indicates more than 200-fold less than random sets. (A) Indirect interactions among the tissue-ubiquitous proteins or among each set of tissue-specific proteins in each of 15 adult tissues. (B) Indirect interactions among the stage-ubiquitous proteins or among each set of stage-specific proteins for each of 30 developmental stages. [file 1471-2105-15-177-S4.pdf]

**A**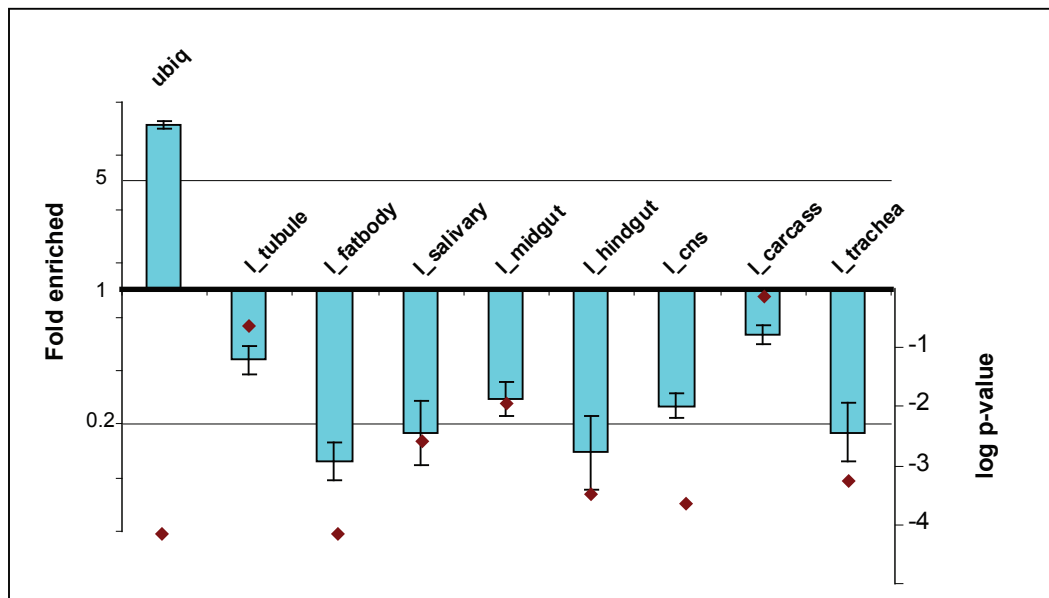**B**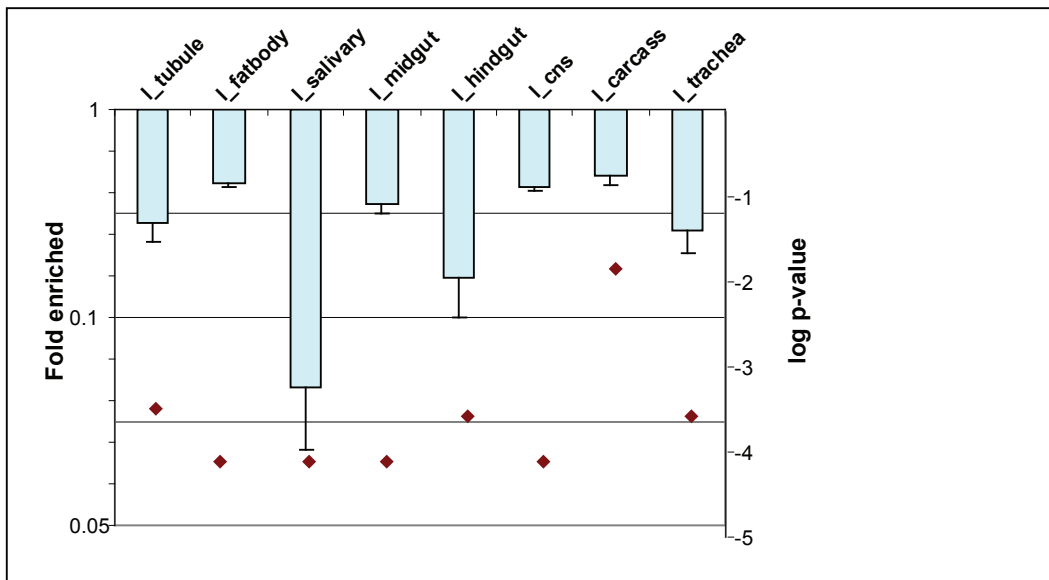

Supplement: Additional file 5 — Within larval tissue subnetworks, tissue-specific proteins rarely interact with each other directly or indirectly. For each protein set the average fold difference between the number of direct (A) or indirect (B) interactions in the test set and number of direct or indirect interactions among proteins in each of 5000 random sets of proteins expressed in the relevant tissue is shown. Standard deviations are shown as error bars. The log p-values for each comparison are shown as red dots (right axis). (A) Direct interactions among the larval tissue-ubiquitous proteins or among each set of larval tissue-specific proteins. (B) Indirect interactions among each set of larval tissue-specific proteins. [file 1471-2105-15-177-S5.pdf]

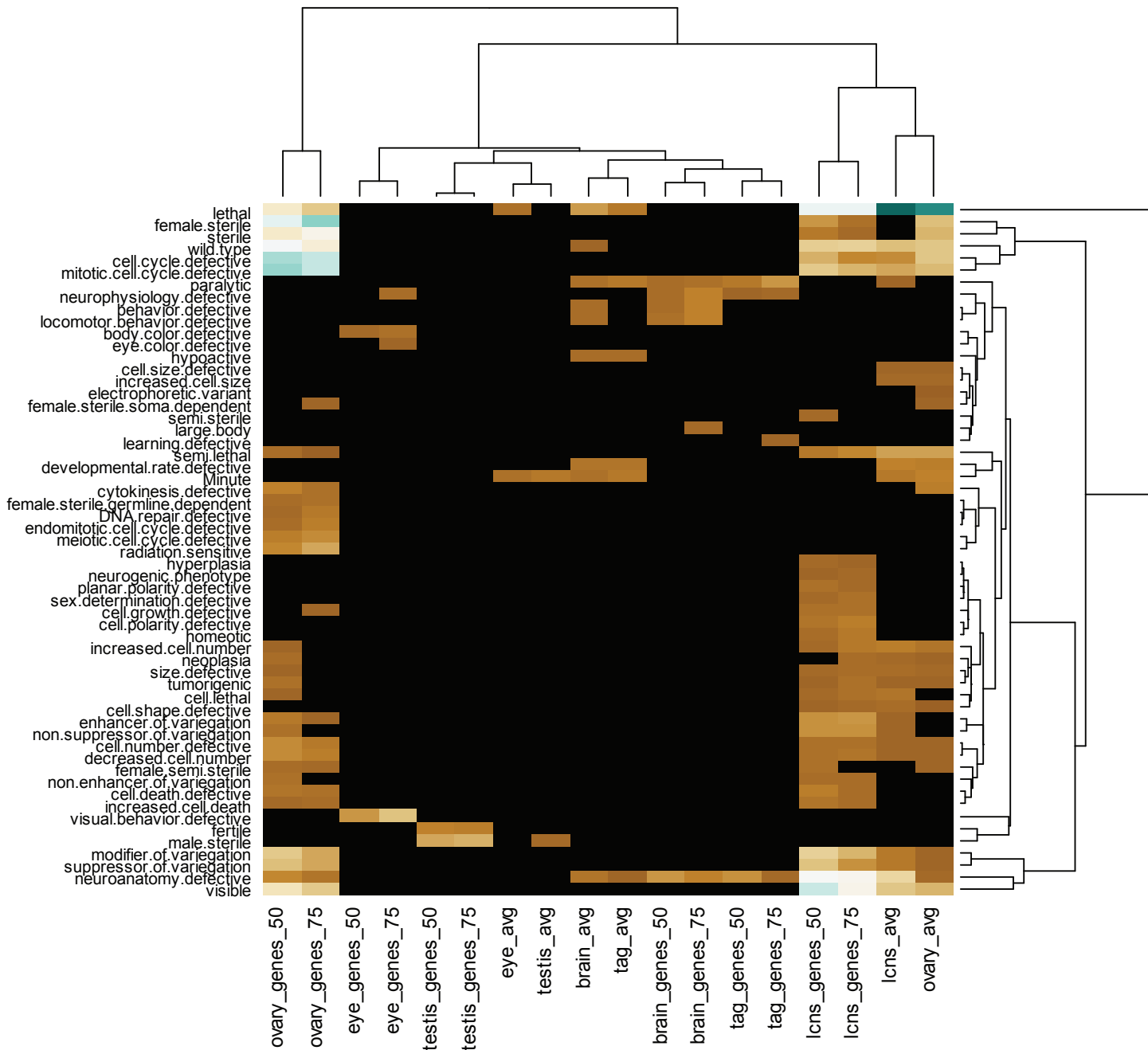

0.05 10e-27

Supplement: Additional file 8 — Heat map of enriched phenotypes in variously filtered gene lists. Heat map of enriched mutant phenotypes obtained in lists of genes because they were expressed above the average, greater than 50 pmax, or greater than 75 pmax in six different tissues. The corrected p-values for enrichment were log transformed, scaled, and then plotted. [file 1471-2105-15-177-S8.pdf]

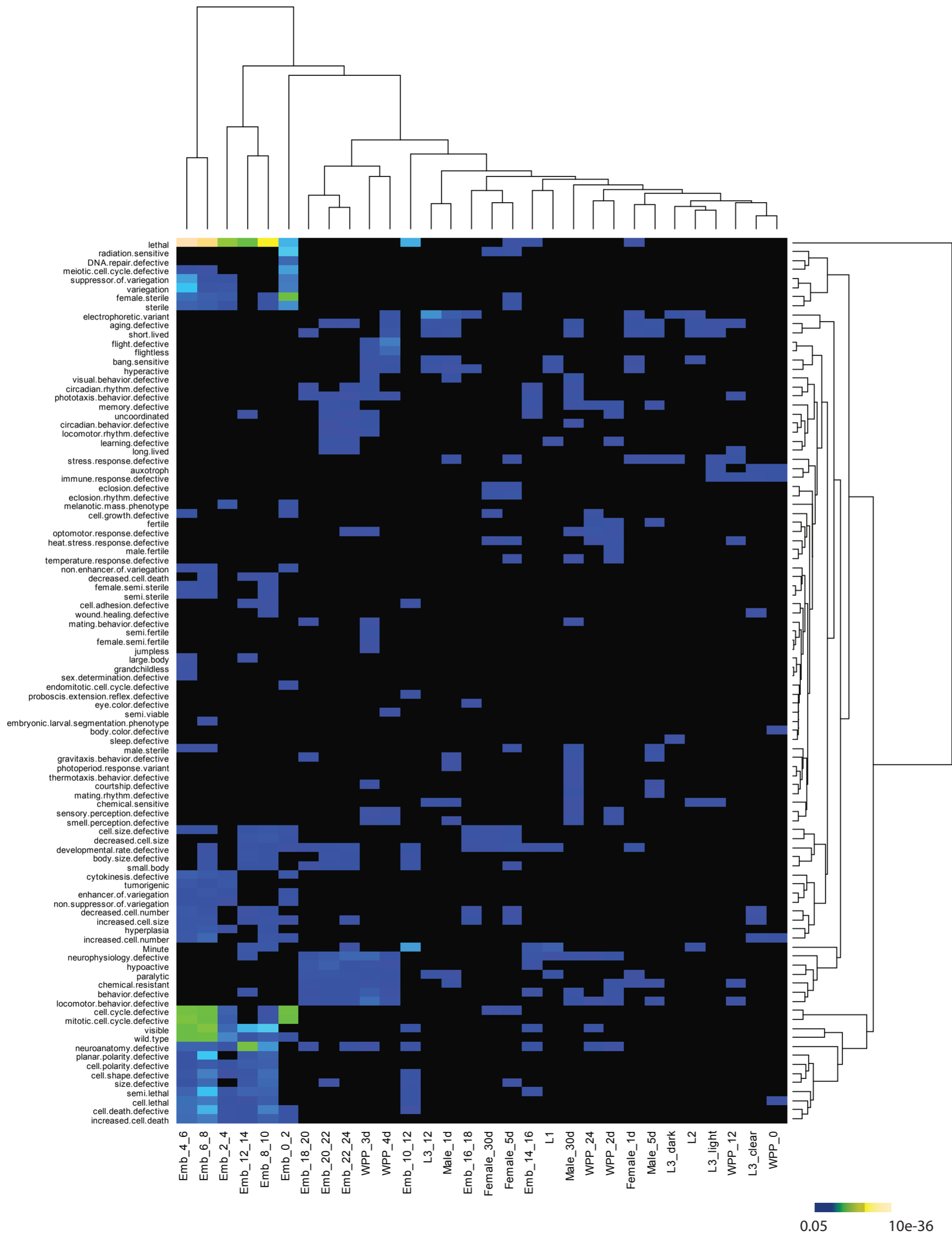

Supplement: Additional file 10 — Heat map of phenotypes enriched in stage-relevant subnetworks. Heat map of enriched mutant phenotypes for genes in subnetworks containing only genes expressed above 75 pmax in each indicated stage. The corrected p-values for enrichment were log transformed, scaled, and then plotted. Stages are clustered based on the similarity of their enriched mutant phenotypes. [file 1471-2105-15-177-S10.pdf]

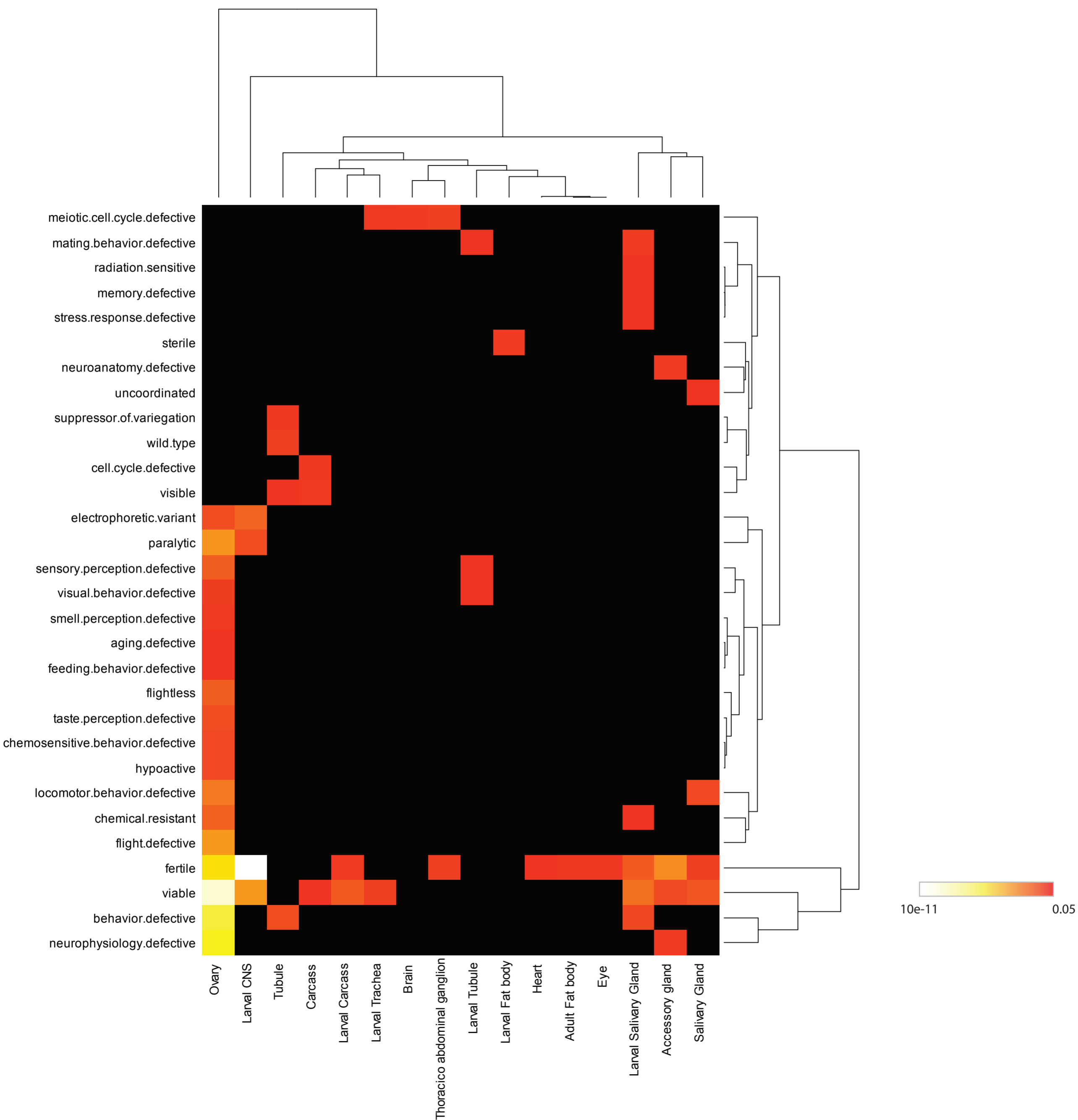

Supplement: Additional file 11 — Heat map of depleted phenotypes in tissue-relevant subnetworks. Heat map of depleted mutant phenotypes for genes in subnetworks containing only genes expressed above 75 pmax in each indicated tissue. The corrected p-values for depletion were log transformed, scaled, and then plotted. [file 1471-2105-15-177-S11.pdf]

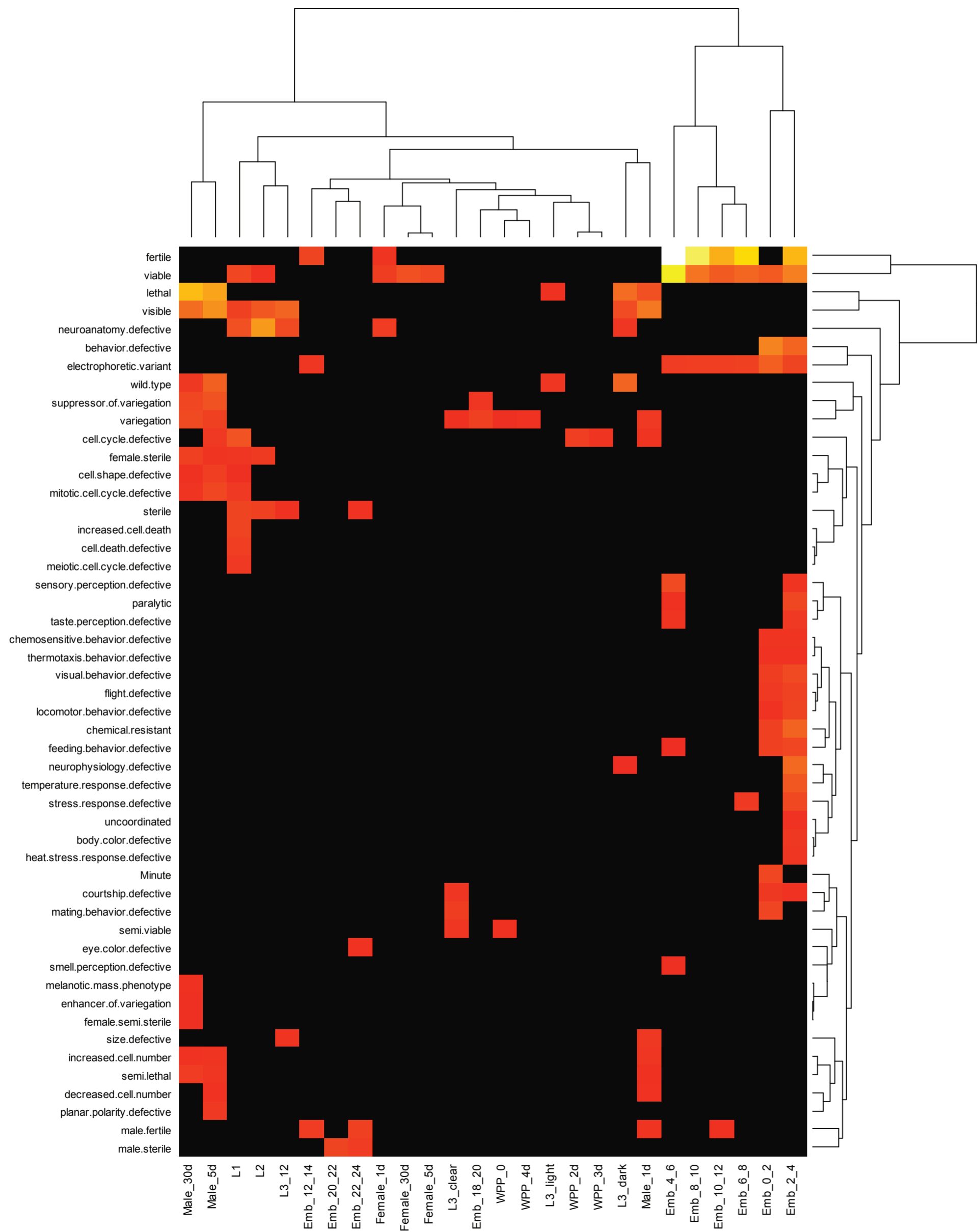

Supplement: Additional file 12 — Heat map of depleted phenotypes in stage-relevant subnetworks. Mutant phenotypes for genes in subnetworks containing only genes expressed above 75 pmax in each indicated stage. The corrected p-values for depletion were log transformed, scaled, and then plotted. [file 1471-2105-15-177-S12.pdf]

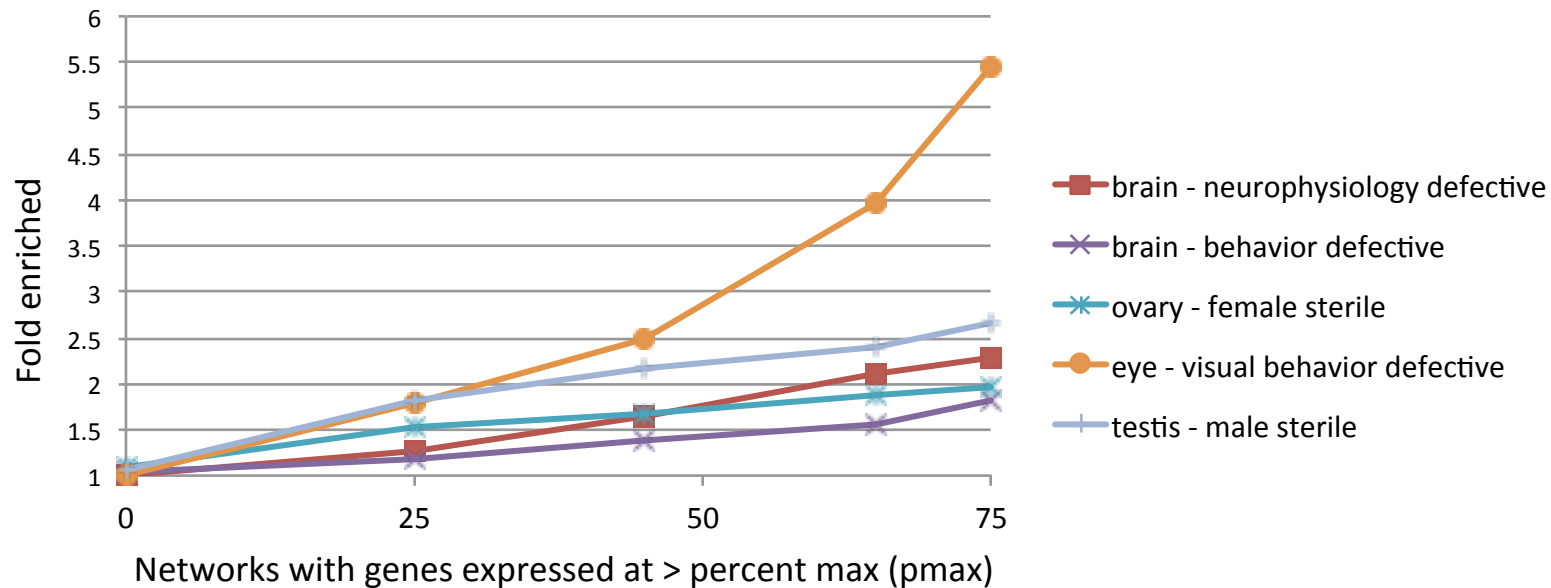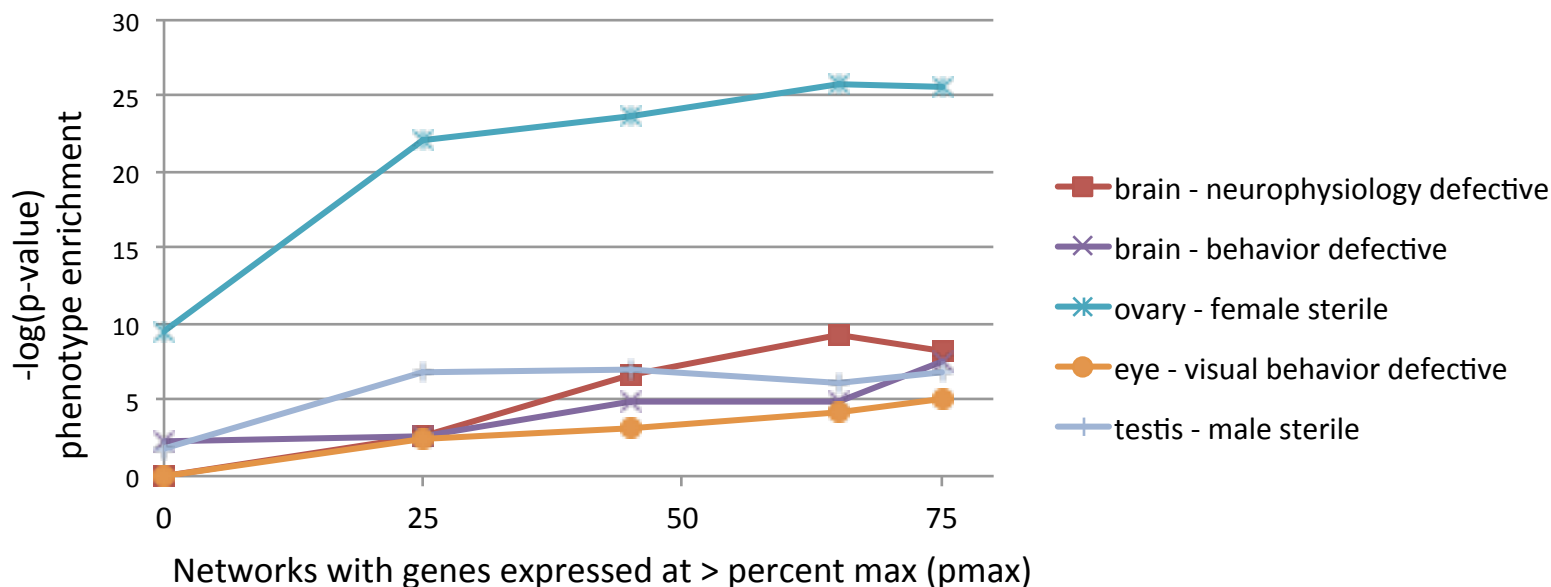

Supplement: Additional file 13 — Networks filtered for genes expressed at higher pmax values are more enriched for context-relevant genes. Top panel: shows fold enrichment for genes with the indicated phenotypes in protein interaction networks filtered for genes expressed above 25, 45, 65, or 85 pmax in brain (neurophysiology defective and behavior defective), ovary (female sterile), eye (visual behavior defective), or testis (male sterile). Fold enrichment is relative to the frequency of finding genes with those phenotypes in the unfiltered network. Bottom panel: Bonferroni-corrected p values for enrichment of the indicated phenotypes in the filtered networks relative to the frequency of those phenotypes in the proteome. Note that the unfiltered network is enriched for genes with some phenotypes, yet the enrichment increases with higher pmax filters. [file 1471-2105-15-177-S13.pdf]
