# Supplementary material for: Integrating the interactome and the transcriptome of Drosophila
Source: BMC Bioinformatics. 2014 Jun 10;15:177. doi: 10.1186/1471-2105-15-177 (PMC4229734; doi:10.1186/1471-2105-15-177)
Supplement: Additional file 6 — Tissue- and stage-specific transcription factors (TFs) regulate tissue- and stage-specific targets in the PDI networks more than expected by chance. For specific TFs in each tissue or stage 100,000 random networks were built by assigning random targets while keeping the node degree constant. P-values (binom.confint, CI = 0.95) were computed by counting the number of times specific interactions in the random networks were lower than the number of specific interactions in the tissue and stage PDI networks. The protein-DNA interaction network (PDI) and the predicted PDI network (Methods) were analyzed separately. [file 1471-2105-15-177-S6.pdf]

Tissue	PDI	predicted PDI
**brain**	0.03	4.67E-05
**crop**	-	9.70E-04
**midgut**	-	0.11
**hindgut**	-	0.76
**ovary**	0.17	0.05
**testis**	1	1.92E-05
**accessory gl**	0.99	0.12
**thoracic gang**	0.17	6.00E-03
**salivary gl**	1	0.25
**eye**	-	0.21
**heart**	0.04	0.93
**0-2 hr embryo**	0.93	0.13
**2-4 hr embryo**	1.92E-05	1.92E-05
**4-6 hr embryo**	1.92E-05	1.92E-05
**6-8 hr embryo**	1.00E-03	1.92E-05
**8-10 hr embryo**	0.5	1.50E-04
**14-16 hr embryo**	-	1.92E-05
**16-18 hr embryo**	-	1.92E-05
**18-20 hr embryo**	0.05	1.92E-05
