# Supplementary material for: Integrating the interactome and the transcriptome of Drosophila
Source: BMC Bioinformatics. 2014 Jun 10;15:177. doi: 10.1186/1471-2105-15-177 (PMC4229734; doi:10.1186/1471-2105-15-177)
Supplement: Additional file 7 — Number of genes in the tissue gene lists after applying different expression filters. Number of genes expressed at average, greater than 50 pmax and greater than 75 pmax in six different tissues. [file 1471-2105-15-177-S7.pdf]

gene_list	# brain	# ovary	# testis	# thoracic gang	# larval CNS	# eye
**average**	2020	1907	2119	1732	2211	1462
**pmax 50**	2754	3469	2704	2437	2984	1598
**pmax 75**	1525	2502	2277	1087	1532	703
**total expressed**	7527	6452	8148	7124	7598	7222
