# Supplementary material for: Integrating the interactome and the transcriptome of Drosophila
Source: BMC Bioinformatics. 2014 Jun 10;15:177. doi: 10.1186/1471-2105-15-177 (PMC4229734; doi:10.1186/1471-2105-15-177)
Supplement: Additional file 9 — Heat map of phenotypes enriched in tissue-relevant subnetworks. Heat map of enriched mutant phenotypes for genes in subnetworks containing only genes expressed above 75 pmax in each indicated tissue. The corrected p-values for enrichment were log transformed, scaled, and then plotted. Tissues are clustered based on the similarity of their enriched mutant phenotypes. [file 1471-2105-15-177-S9.pdf]

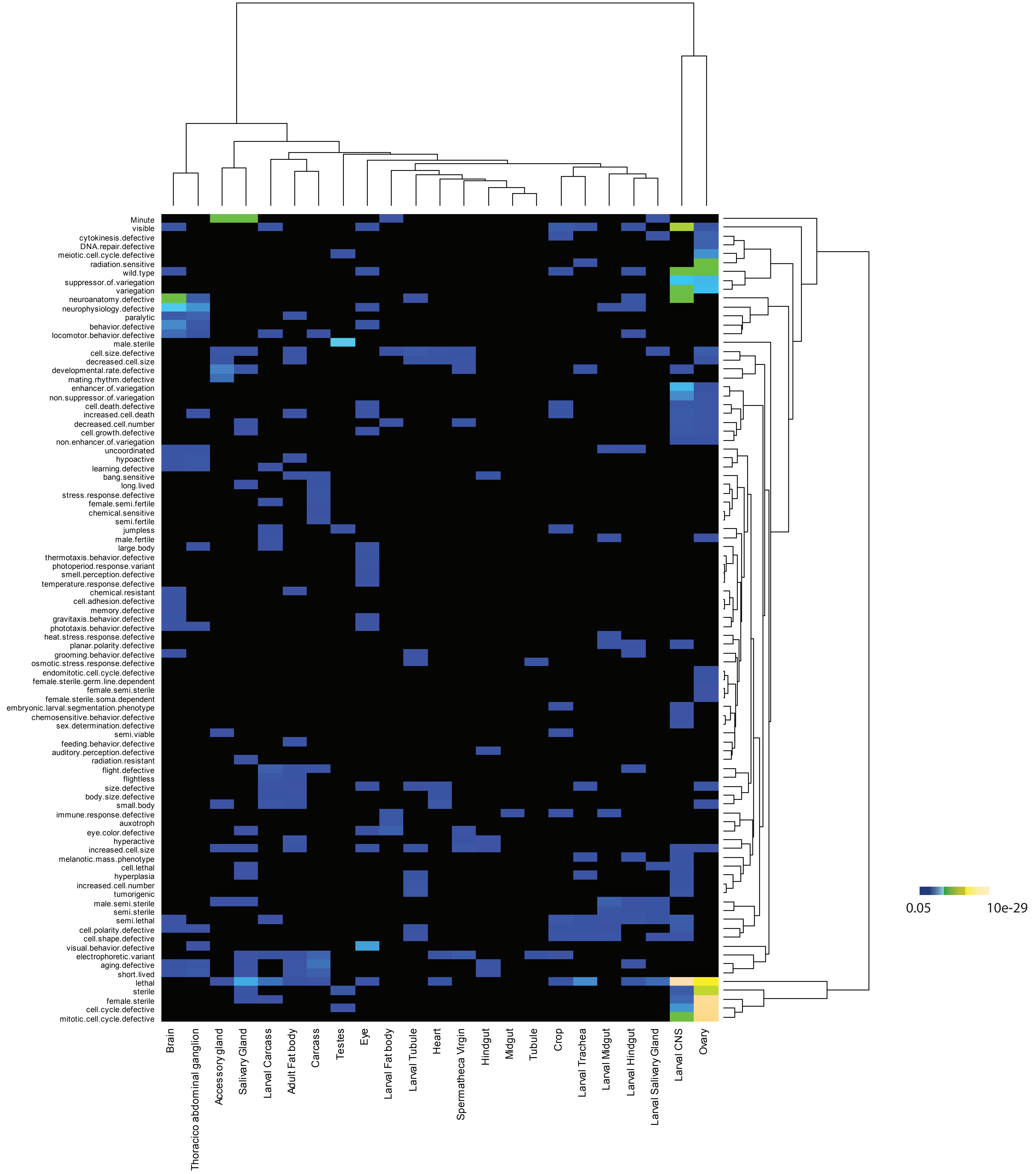

Minute
visible
cytokinesis.defective
DNA.repair.defective
meiotic.cell.cycle.defective
radiation.sensitive
wild.type
suppressor.of.variegation
variegation
neuroanatomy.defective
neurophysiology.defective
paralytic
behavior.defective
locomotor.behavior.defective
male.sterile
cell.size.defective
decreased.cell.size
developmental.rate.defective
mating.rhythm.defective
enhancer.of.variegation
non.suppressor.of.variegation
cell.death.defective
increased.cell.death
decreased.cell.number
cell.growth.defective
non.enhancer.of.variegation
uncoordinated
hypoactive
learning.defective
bang.sensitive
long.lived
stress.response.defective
female.semi.fertile
chemical.sensitive
semi.fertile
jumpless
male.fertile
large.body
thermotaxis.behavior.defective
photoperiod.response.variant
smell.perception.defective
temperature.response.defective
chemical.resistant
cell.adhesion.defective
memory.defective
gravitaxis.behavior.defective
phototaxis.behavior.defective
heat.stress.response.defective
planar.polarity.defective
grooming.behavior.defective
osmotic.stress.response.defective
endomitotic.cell.cycle.defective
female.sterile.germ.line.dependent
female.semi.sterile
female.sterile.soma.dependent
embryonic.larval.segmentation.phenotype
chemosensitive.behavior.defective
sex.determination.defective
semi.viable
feeding.behavior.defective
auditory.perception.defective
radiation.resistant
flight.defective
flightless
size.defective
body.size.defective
small.body
immune.response.defective
auxotroph
eye.color.defective
hyperactive
increased.cell.size
melanotic.mass.phenotype
cell.lethal
hyperplasia
increased.cell.number
tumorigenic
male.semi.sterile
semi.sterile
semi.lethal
cell.polarity.defective
cell.shape.defective
visual.behavior.defective
electrophoretic.variant
aging.defective
short.lived
lethal
sterile
female.sterile
cell.cycle.defective
mitotic.cell.cycle.defective
Brain
Thoracico abdominal ganglion
Accessory gland
Salivary Gland
Larval Carcass
Adult Fat body
Carcass
Testes
Eye
Larval Fat body
Larval Tubule
Heart
Spermatheca Virgin
Hindgut
Midgut
Tubule
Crop
Larval Trachea
Larval Midgut
Larval Hindgut
Larval Salivary Gland
Larval CNS
Ovary
0.05
10e-29
